# Inactivation of the *Burkholderia* Toxin Malleicyprol by Enzymatic Cyclopropanol Ring Opening

**DOI:** 10.1002/anie.202521105

**Published:** 2025-11-10

**Authors:** Jonas Fiedler, Ingrid Richter, Katharina Dornblut, Alicia Scharf, Christian Hertweck

**Affiliations:** ^1^ Department of Biomolecular Chemistry Leibniz Institute for Natural Product Research and Infection Biology (HKI) Beutenbergstraße 11a 07745 Jena Germany; ^2^ Unidad de Genómica Avanzada Centro de Investigación y de Estudios Avanzados del Instituto Politécnico Nacional Km 9.6 Libramiento Norte Carretera IrapuatoLeón Irapuato Guanajuato CP36824 Mexico; ^3^ Natural Product Chemistry, Faculty of Biological Sciences Friedrich Schiller University Jena 07743 Jena Germany; ^4^ Cluster of Excellence Balance of the Microverse Friedrich Schiller University Jena 07743 Jena Germany

**Keywords:** Biosynthesis, Cleavage reactions, Oxidoreductases, Redox chemistry, Virulence factor

## Abstract

Pathogenic bacteria of the *Burkholderia pseudomallei* group cause life‐threatening infections in humans and animals. Their virulence factors include malleicyprols bearing a reactive cyclopropanol moiety essential for toxicity. Inactivating this reactive motif, therefore, is a promising way to neutralize these toxins. Here, we identify a heme‐dependent oxidoreductase (BurK) that cleaves the cyclopropanol warhead. Mutational analyses and in vivo radical capturing show that BurK catalyzes a radical ring opening to yield a propanone fragment. Characterizing BurK orthologs across various bacterial phyla suggests broader ecological roles of these unusual enzymes. Using a nematode model, we demonstrate that BurK‐producing helper bacteria neutralize malleicyprols, significantly reducing toxicity and enhancing host survival. In addition to uncovering a novel biocatalyst, this work lays the foundation for antivirulence approaches using therapeutic microbes against antibiotic‐resistant pathogens.

## Graphical abstract

*Burkholderia pseudomallei* and *Burkholderia mallei* are dangerous pathogens that cause severe diseases with high mortality rates. Their virulence relies in part on malleicyprols, potent toxins containing a highly reactive cyclopropanol group. In this study, we identify BurK, a heme‐dependent oxidoreductase that neutralizes malleicyprols by enzymatically opening the cyclopropanol ring.

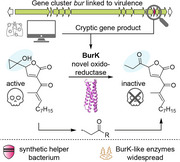

## Introduction

*Burkholderia pseudomallei* and *Burkholderia mallei* are closely related Gram‐negative bacteria known for causing severe and often fatal diseases in humans and animals. *B. pseudomallei* is the causative agent of melioidosis, a neglected tropical disease with approximately 165 000 annual cases and a mortality rate approaching 50% even with antibiotic treatment.^[^ ^1^, ^2^ ^]^ Its close relative, *B. mallei*, induces glanders in horses and was infamously weaponized during World War I.^[^ ^3^ ^]^ Both pathogens are highly antibiotic‐resistant, and no vaccine is currently available, underscoring the urgent need for alternative therapeutic strategies.^[^ ^4^ ^]^ One promising approach is the antivirulence therapy, which targets pathogenicity factors rather than bacterial viability.^[^ ^5^ ^]^ However, developing such interventions requires a detailed mechanistic understanding of the molecular basis of pathogenicity. The virulence of bacteria of the *B. pseudomallei* group relies in part on a complex of specialized metabolites named malleicyprols,^[^ ^6^ ^]^ which includes malleicyprol (**1**), bis‐malleicyprol (**2**), and congeners with various side chains.^[^ ^6^, ^7^ ^]^ The toxic effects of these compounds are attributed to the presence of a cyclopropanol moiety.^[^ ^6^ ^]^ In contrast, the related constitutional isomer, burkholderic acid (syn. malleilactone) (**3**),^[^ ^8^, ^9^ ^]^ which features a propanone side chain in lieu of the cyclopropanol, has been shown to be inactive in assays against both an animal model (the nematode *Caenorhabditis elegans*) and mammalian cell lines.^[^ ^6^, ^9^ ^]^ Therefore, inactivating malleicyprols or inhibiting their production by pathoblockers represents promising avenues to disarm these severe pathogens.

**Figure 1:**
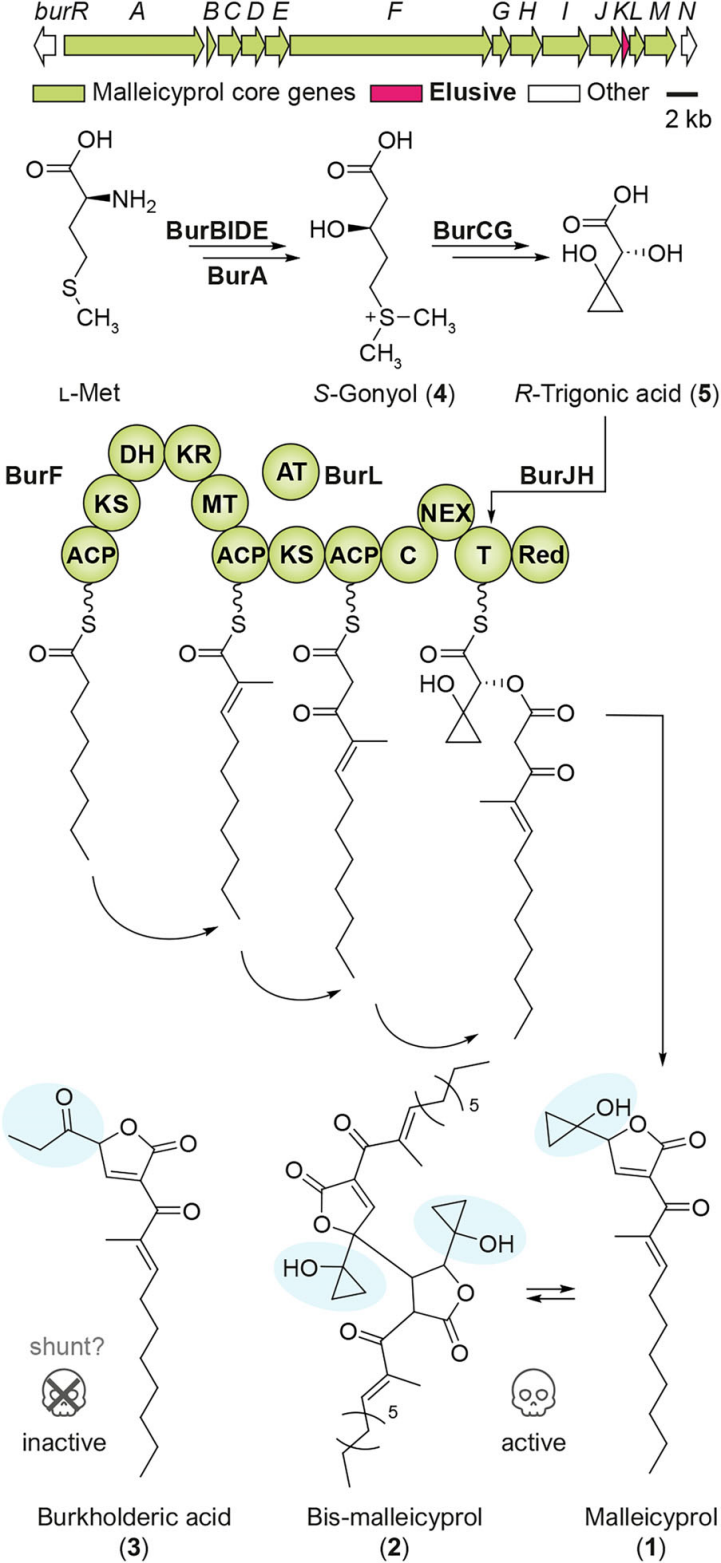
Architecture of the *bur* gene cluster and model of malleicyprol biosynthesis. The biosynthetic steps leading to **1** and **2** have been fully characterized. The function of BurK has been elusive. Malleicyprol and bis‐malleicyprol are toxic against nematodes and show antiproliferative effects against mammalian cell lines. Burkholderic acid is inactive against nematodes or mammalian cell lines and was believed to be a spontaneous degradation product from **1** (a more detailed scheme is shown in Figure S1) M: module; ACP: acyl carrier protein; KS: ketosynthase; DH: dehydratase; KR: ketoreductase; MT: methyltransferase; AT: acyltransferase; C: condensation domain; T: thiolation domain; Red: reductase; NEX: N‐terminal extension.

Analyses at the genetic, biochemical, and chemical levels have provided insight into the enzymatic assembly of malleicyprol and identified potential enzymatic targets for an antivirulence therapy. All malleicyprol biosynthesis enzymes are encoded in a 38 kb gene cluster designated *bur* (syn. *mal*), which is highly conserved in bacteria of the *B. pseudomallei* group (Figure 1).^[^ ^6^, ^9^, ^10^ ^]^ The biosynthetic pathway is initiated by conversion of l‐methionine to the sulfonium acid dimethylsulfoniumpropionate (DMSP) by a set of four enzymes (BurBIDE).^[^ ^11^ ^]^ The PKS‐NRPS hybrid BurA elongates DMSP with malonyl‐CoA to produce *S*‐gonyol (**4**).^[^ ^11^ ^]^ *S*‐gonyol is then transformed into *R*‐trigonic acid (**5**) by means of a hydroxylase (BurC) and an unusual ketol‐acid reductoisomerase‐like enzyme (BurG).^[^ ^12^ ^]^ The α‐hydroxy acid **5** is then loaded onto the PKS‐NRPS hybrid BurF by BurJ and BurH,^[^ ^13^ ^]^ and fused to an independently formed fatty acid‐polyketide hybrid generated by BurF.^[^ ^7^ ^]^ Reductive release and cyclization gives **1**, which is in equilibrium with the spontaneously formed dimer **2** (see Figure S1 for a more detailed biosynthesis scheme).^[^ ^6^, ^14^ ^]^ The propanone‐substituted isomer **3** was believed to be a spontaneous degradation product from **1**.^[^ ^15^ ^]^

Although the biosynthetic steps that lead to the malleicyprol complex have been elucidated, the function of the gene product of *burK*, which has been annotated as lipoprotein,^[^ ^10^ ^]^ has remained obscure. Here, we report that BurK is a heme‐dependent oxidoreductase that inactivates malleicyprol toxins by opening the cyclopropanol ring through formation of a β‐keto radical intermediate. In addition, by developing a protection assay using a nematode model, we demonstrate that in vivo expression of BurK reduces malleicyprol toxicity and enhances host survival, thus providing a foundation for the development of antivirulence strategies against this serious pathogen.

## Results and Discussion

To gain insight into the role and potential function of *burK*, we first analyzed a published transcriptomic dataset^[^ ^16^ ^]^ on the response of *B. pseudomallei* to different niches and noted that *burK* is upregulated during host infection (Table S1). In addition, we analyzed data from an independent study in which transposon insertion and sequencing (Tn‐seq) was used to identify genes required for in vivo fitness in a mouse model.^[^ ^17^ ^]^ We observed that the transposon‐mediated disruption of *burK* leads to a substantially reduced abundance of these strains after lung colonization compared to the initial population. Notably, *burK* disruption was the most impactful alteration from the *bur* gene cluster and ranks among the top 2% of genes with the highest fold‐change, according to the dataset (Table S2).

**Figure 2:**
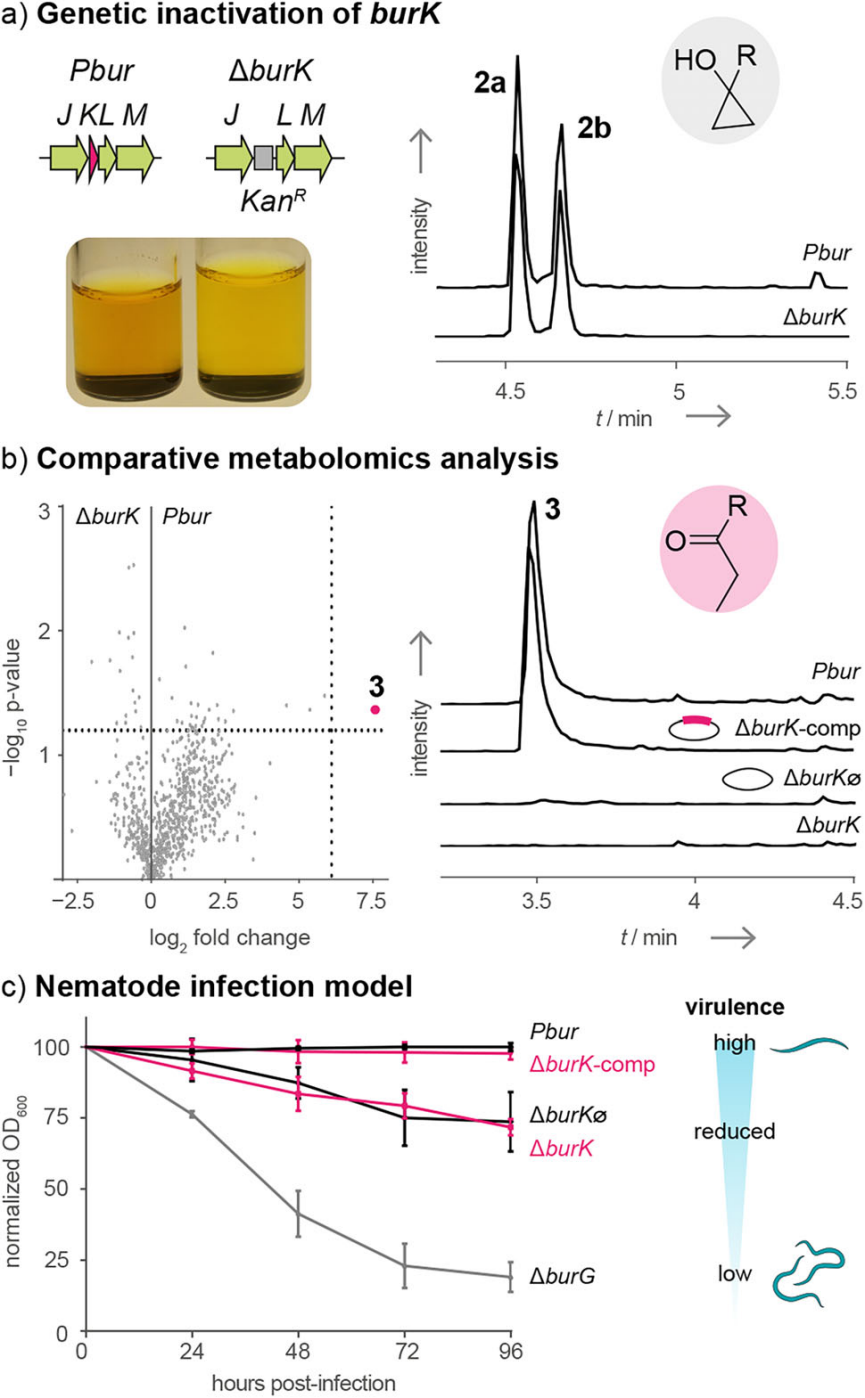
Genetic inactivation of *burK* and physiological investigations. a) The gene *burK* was inactivated by introduction of a kanamycin resistance cassette by homologous recombination. The extract of the resulting Δ*burK* strain is light yellow compared to the dark yellow color of extracts of the overproducing strain *Pbur*. The dimer **2** is still detected by HPLC‐HRMS analysis observed as mixture containing two main diastereomers (extracted ion chromatogram (EIC): *m*/*z* 611.3589; [M − H]^−^). b) A comparative metabolomics analysis (*Pbur* versus Δ*burK*) revealed that the isomer **3** (EIC: *m*/*z* 305.1758; [M − H]^−^) is absent in the mutant strain. c) *C. elegans* was co‐incubated with *B. thailandensis Pbur*, Δ*burK*, Δ*burG*, complemented Δ*burK* (Δ*burK*‐comp), or the empty‐plasmid control (Δ*burK*∅) as food source. The number of viable nematode worms in the suspension is directly related to the bacterial cell density. OD_600_ values obtained from wells with nematodes were normalized against values from wells without nematodes. Normalized mean OD_600_ values from three independent experiments (*n* = 3 biological replicates) were plotted as a percentage of the initial OD_600_ ± 1 standard error of mean (SEM). Absence of BurK (Δ*burK*) leads to reduced virulence compared to the overproducing strain (*Pbur*). Kan^R^: kanamycin resistance cassette.

These findings prompted us to investigate the role of the obscure gene product, BurK, in virulence. Therefore, we used the *Burkholderia thailandensis* *Pbur* (*Pbur*) strain, an established low‐virulence model organism, which constitutively expresses the *bur* gene cluster.^[^ ^10^ ^]^ To inactivate *burK*, we constructed the plasmid pJET‐*burK* with a kanamycin resistance cassette between homology regions flanking the target gene (Figure 2a). After introducing this plasmid into the *Pbur* strain and verifying the successful homologous recombination by polymerase chain reaction (Figure S2), we cultured the resulting mutant strain *Pbur*Δ*burK (*Δ*burK*). In contrast to the dark yellow extracts typical of *Pbur* strain cultures (Figure 2a), the culture extract of the mutant appeared light yellow. This phenotype has been associated with the abolishment of malleicyprol biosynthesis.^[^ ^6^, ^7^, ^11^, ^13^ ^]^ However, analysis of the culture extract by high‐performance liquid chromatography‐high‐resolution mass spectrometry (HPLC‐HRMS) showed that **1** and **2** (*m*/*z* 611.3589; [M − H]^−^) were still produced (Figure 2a) in similar amounts as in the *Pbur* strain. Nevertheless, when we performed a comparative metabolomic analysis (*Pbur* versus ΔburK), we found the specific absence of the inactivated isomer **3** (*m*/*z* 305.1758; [M − H]^−^) in the mutant strain (Figure 2b).

To exclude the possibility of polar effects caused by the gene disruption, we conducted a genetic complementation by introducing the pSCrhaB2‐based^[^ ^18^ ^]^ expression plasmid pSCrhaB2‐*burK* into the Δ*burK* strain, generating Δ*burK*‐comp (Figure S3). Co‐expression of *burK* restored the formation of **3** (Figure 2b) in contrast to the empty‐plasmid control strain (Δ*burK*∅). These results demonstrate that BurK is responsible for the conversion of the cyclopropanol‐bearing malleicyprols into the propanone‐substituted **3**.

The function of BurK is reminiscent of the inactivation of colibactin, in which the enzyme ClbS hydrolyzes a cyclopropane moiety as part of self‐resistance mechanisms.^[^ ^19^ ^]^ Previous infection models using the Δ*clbS* strain showed reduced virulence, which was attributed to reduced viability and fitness.^[^ ^20^ ^]^ Therefore, we first tested the viability of the Δ*burK* strain in more detail. In a noninfective context, such as axenic cultures in a liquid medium, both *Pbur* and Δ*burK* exhibit similar growth (Figure S4), and no obvious deficiencies were observed during our studies. Additionally, agar diffusion tests with isolated **2** did not show attenuation of the Δ*burK* strain (Figure S4). Nonetheless, because *burK* is present in all available genomes of potential malleicyprol producers (Figure S5), we assumed that the capacity to transform the malleicyprols into **3** is an important trait for these strains. Based on the previous Tn‐seq study,^[^ ^17^ ^]^ we adapted our physiological studies to reflect an infective context. To do this, we established an infection assay using the nematode *C. elegans*. The model organism was cocultured with bacteria as a food source and the number of viable nematode worms in the suspension was directly related to the bacterial cell optical density at 600 nm (OD_600_). As a positive control, we used the malleicyprol‐overproducing strain *Pbur*, which should readily kill nematodes. The non‐producer (Δ*burG*), which is vulnerable to nematodes, served as the negative control (Figure 2c). When confronted with the nematode population, the Δ*burK* mutant exhibits reduced fitness compared to the *Pbur* strain (Figure 2c). Nevertheless, the malleicyprol complex present in the Δ*burK* strain still causes substantial nematode death (see Figure S6 for microscopy images), compared to the Δ*burG* strain. The full pathogenic capacity and fitness of the bacteria were restored in the Δ*burK*‐comp strain, but not in the Δ*burK*∅ control strain (Figure 2c).

Taken together, these physiological experiments show that BurK is not required when the producer grows axenically but is important in a pathogenic context. Based on the nontoxicity of **3** against the nematode model,^[^ ^9^ ^]^ the results might suggest that the *Pbur* strain regulates the levels of **1** and **2** by BurK to ensure its own full fitness and pathogenic potential during the host–pathogen interactions. This aligns with independent transcriptomic and Tn‐seq studies,^[^ ^16^, ^17^ ^]^ and the decreased virulence of the Δ*clbS* strain.^[^ ^20^ ^]^ We currently cannot exclude that either BurK or **3** might have additional, so far elusive functions in the pathogenic context (e.g., activity of **3** as metallophore^[^ ^9^ ^]^ or as regulatory molecule^[^ ^21^ ^]^).

Apart from these physiological studies, more critically, the identified enzyme BurK represents a tool to enzymatically inactivate the toxins of the malleicyprol complex. In an effort to understand the reaction mechanism of BurK, we performed bioinformatic analyses of its deduced amino acid sequence. Using the ProtParam tool, we found that most residues are hydrophobic (66%) and that the calculated isoelectric point is highly basic (pI: 9.5), which are typical characteristics of membrane proteins (Figure S7).^[^ ^22^, ^23^ ^]^ This assessment was supported by Phyre^2^ and TMHMM analyses (Figure S7),^[^ ^24^, ^25^ ^]^ which predict that BurK is localized in the cytosolic membrane as an integral membrane protein. Thus, unsurprisingly, all attempts to obtain a soluble, isolated protein for in vitro studies were futile. Therefore, we focused on mutational analyses guided by bioinformatics and in vivo studies.

**Figure 3:**
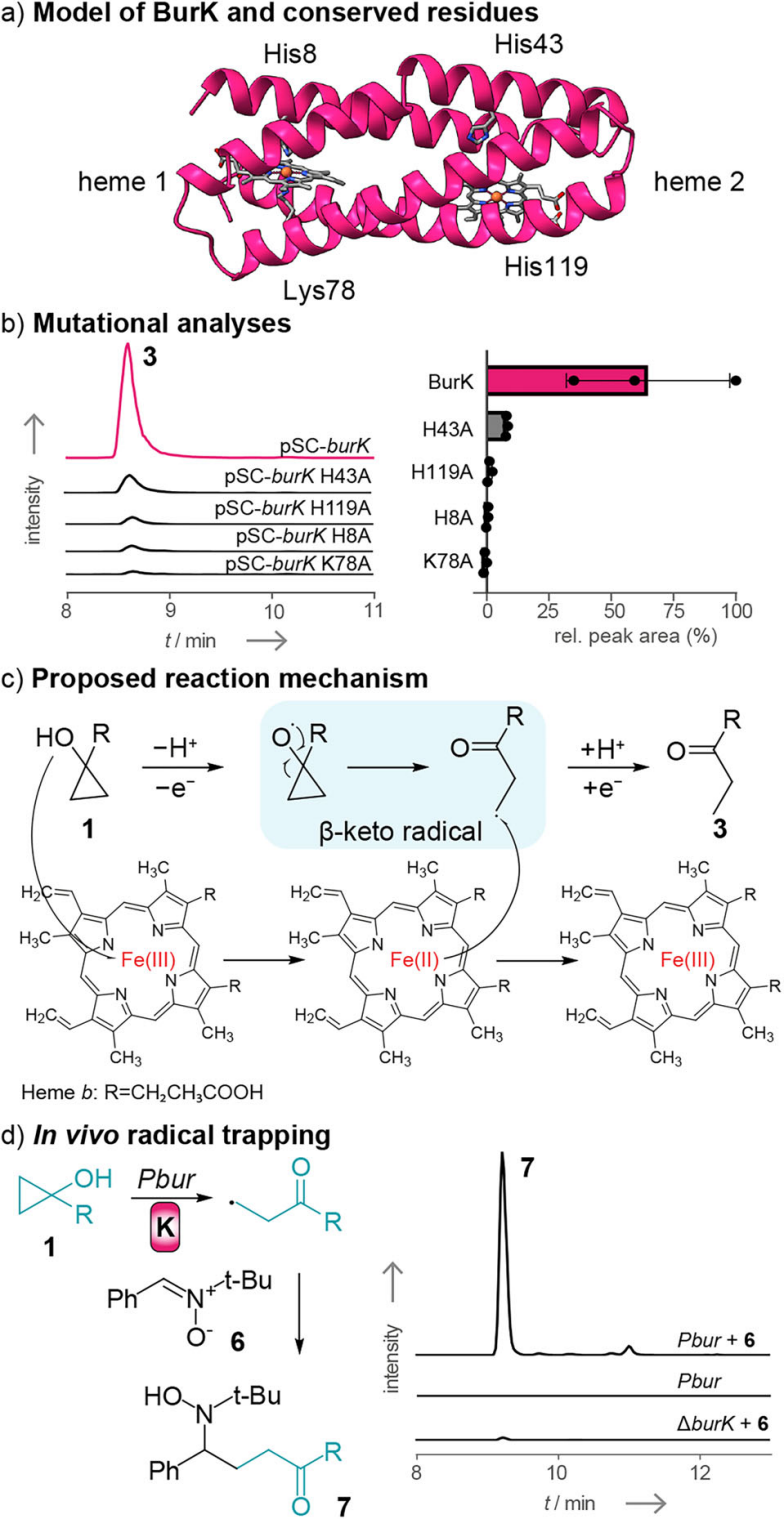
Structural and mutational investigations show that BurK is a novel oxidoreductase. a) Modeling of BurK predicts that the protein adopts a four‐helical fold. Comparison with the superoxide oxidase CypB indicates that BurK contains two heme cofactors (see Supporting Information for details). b) The alanine substitution of key histidine residues either abolishes or significantly reduces the product formation of burkholderic acid (**3**) to background levels (EIC: *m*/*z* 305.1758; [M − H]^−^); *n* = 3 biological replicates. c) The presence of the heme cofactor indicates that the reaction mechanism follows a radical pathway (see Supporting Information for alternative reaction pathway). d) The in vivo radical trapping reagent PBN was used to capture the proposed β‐keto radical. Addition to a *Pbur* culture led to the formation of compound **7** (EIC: *m*/*z* 484.3057; [M + H]^+^).

According to the deduced membrane topology and an AlphaFold3 (AF3) model,^[^ ^26^ ^]^ BurK would adopt a four‐helical bundle structure (Figure 3a) with the *N*‐ and *C*‐termini facing the periplasmic space. An HHPred^[^ ^27^ ^]^ analysis shows that BurK shares high structural similarity with the membrane‐bound superoxide oxidase CypB from *Escherichia* coli (Figure S8).^[^ ^28^ ^]^ This cytochrome *b*‐type enzyme catalyzes the oxidation of periplasmic superoxide (O_2−_) into molecular oxygen (O_2_), coupled to the reduction of cytoplasmic ubiquinone to ubiquinol.^[^ ^28^ ^]^ CypB contains two heme *b* cofactors coordinated by axial histidine residues, located in helices 1 and 3 (heme 1) or helices 2 and 4 (heme 2), respectively.^[^ ^28^ ^]^ By structural analogy, two noncovalently bound heme groups would be embedded within the four‐helical bundle of BurK. Indeed, comparison of the AF3 model of BurK with the CypB structure predicts high congruence and shows that His8, His43, and His119 (based on BurK numbering) are conserved between the two folds (Figure S8). In contrast, BurK contains a lysine residue (Lys78) in place of the histidine found in helix 3 of CypB required for heme 1 coordination. Nonetheless, such lysine residues are also common ligands for heme coordination.^[^ ^29^ ^]^

To shed light on the relevance of these residues in BurK, we generated numerous variants in which the conserved residues were individually substituted by alanine. Expression plasmids with the mutated *burK* variants (e.g., pSCrhaB2‐*burK*‐H8A) were then individually introduced into Δ*burK*, and the resulting strains (e.g., Δ*burK* pSCrhaB2‐*burK*‐H8A) were cultivated and extracted as described before. HPLC‐HRMS analysis of the extracts showed that the formation of **3** is abolished when His8, Lys78, or His119 are mutated, and only minute amounts of **3** could be detected in the His43Ala mutant (Figure 3b). These results suggest that BurK contains one heme *b* cofactor coordinated by His8 and Lys78. Based on drastically reduced product formation in the His43Ala mutant and the absence of detectable product in the His119Ala mutant, also a second heme *b* is expected, as found in the homologous protein CypB.

The ring‐opening of cyclopropanol moieties in synthetic chemistry typically proceeds via a radical pathway.^[^ ^30^ ^]^ A heme‐coordinated Fe(III) is well‐suited to act as an oxidizing agent for the cyclopropanol unit of **1**, generating a β‐keto radical intermediate, while being reduced to the ferrous Fe(II) form. It is important to note that during the oxidation step, the generated radical could initially be either oxygen‐centered at the cyclopropanol moiety (Figure 3c, analogous to mechanisms proposed for synthetic cyclopropanol‐containing reactions^[^ ^30^ ^]^) or carbon‐centered at the γ‐carbon atom of the butenolide ring (Figure S9, as observed for some cyclopropane reactions^[^ ^30^ ^]^), both ultimately leading to the same β‐keto radical intermediate. Electron return would then quench the β‐keto radical, resulting in the formation of **3** and restoring the initial Fe(III) oxidation state. During these steps, a proton must be abstracted from **1** and later donated to the β‐keto radical intermediate. A protein–ligand co‐folding model, generated by Boltz‐2,^[^ ^31^ ^]^ predicts that **1** could fit in a negatively charged pocket next to the heme 2 cofactor (Figure S10). There, a glutamic acid residue may also facilitate proton shuttling. The second heme 1 cofactor would be unrelated to catalysis in the proposed reaction sequence but could be required for structural integrity of the protein, as indicated by the mutagenesis analysis.

**Figure 4:**
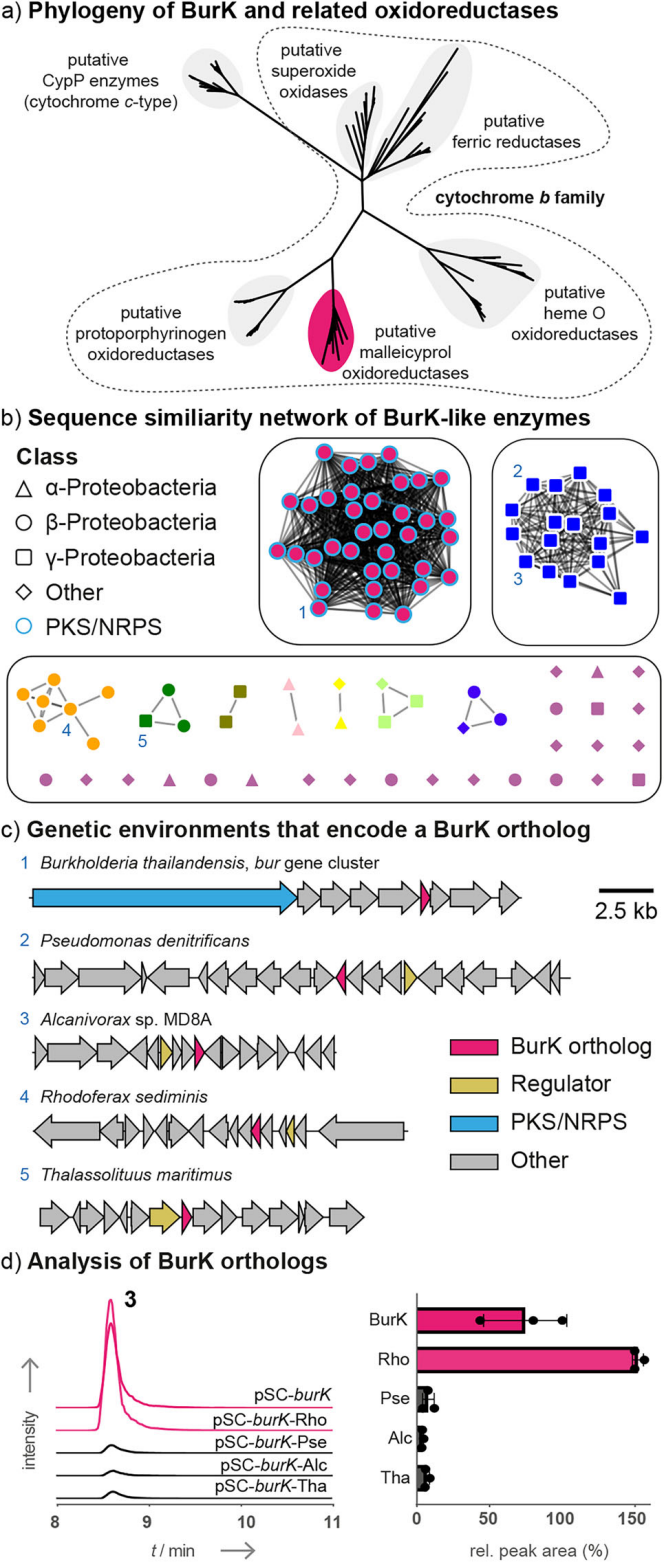
Identification and analysis of BurK‐like enzymes show that they are widely distributed. a) Phylogenetic analysis of BurK and related oxidoreductases. See Figure S12 for a more detailed representation. b) Sequence similarity network of BurK homologs. c) Genetic environments of *burK*‐like genes in comparison to the *bur* gene cluster. d) Cross‐complementation of the *Pbur*Δ*burK* strain with *burK*‐like genes leads to the formation of **3** (EIC: *m*/*z* 305.1758; [M − H]^−^) and implies a role in an environmental context.

To test the model of a radical‐based cyclopropanol ring opening, we aimed to capture the proposed β‐keto radical intermediate. Therefore, we supplemented *Pbur* cultures with the commonly used in vivo radical‐trapping agent *N*‐*tert*‐butyl‐α‐phenylnitrone (**6**) (PBN).^[^ ^32^ ^]^ Indeed, HPLC‐HRMS^2^ analysis of *Pbur* culture extracts showed the formation of a new compound (*m*/*z* 484.3057; [M + H]^+^). Both the exact mass and the MS^2^ fragmentation pattern^[^ ^33^ ^]^ pointed to the expected PBN adduct **7** (Figure S11). As negative controls, we examined extracts of *Pbur* cultures without **6**, and Δ*burK* cultures supplemented with **6**. As expected, neither of the negative controls showed the formation of the PBN compound (Δ*burK +* **6**: <1% of rel. peak area, attributed to spontaneous radical formation). These findings provide additional support for the proposed radical mechanism of BurK. Furthermore, the radical capture suggests that the β‐keto radical may leave the enzyme and react with various targets. Based on sequence relations of BurK with other oxidoreductases (Figures 4a and S12), our mutational analyses, and the PBN‐biotransformation experiment, we propose that BurK functions as malleicyprol oxidoreductase of the diverse cytochrome *b* enzyme family.

To determine whether further malleicyprol oxidoreductases (BurK orthologs) are present in other bacteria, we constructed a sequence similarity network using EFI‐EST^[^ ^34^ ^]^ (Figure 4a). The majority (37%) of orthologs are encoded in putative *bur* gene clusters of *Burkholderia* species. The other *burK*‐like genes are not co‐localized with genes typically associated with specialized metabolism, but they share the feature that regulatory proteins are encoded in their genomic neighborhoods (Figure 4b). To test the potential roles of the BurK relatives, we selected four corresponding genes from main hubs of the network, specifically from *Pseudomonas denitrificans* (Pse), *Alcanivorax* sp. MD8A (Alc), *Rhodoferax sediminis* (Rho), and *Thalassolituus maritimus* (Tha) (see Table S3). For each gene, we constructed pSCrhaB2 expression plasmids (e.g., pSCrhaB2‐*burK*‐Pse), which were then individually introduced into the Δ*burK* strain for cross‐complementation experiments. The generated strains (e.g., Δ*burK* pSCrhaB2‐*burK*‐Pse) were then cultivated and extracted as described before. HPLC‐HRMS analysis showed that all orthologs promote the formation of **3**, albeit at different rates (Figure 4c). Interestingly, the ortholog from *R. sediminis* exceeds the titers of **3** in comparison to native BurK. In contrast, the other three orthologs generate substantially lower amounts of **3** (∼10% product formation compared to BurK).

A comparison of the primary sequences and AF3 models of the tested BurK‐like proteins (Figure S13) revealed that the residues His8, His43, and Lys78 are conserved in all proteins and that the overall fold adopts the expected four‐helical fold. However, the His119 residue of BurK is absent in BurK‐Pse and BurK‐Alc and differently positioned in BurK‐Tha. According to the structure model, BurK‐Rho contains the His119 residue in the same orientation as native BurK. These traits might explain the differences in product formation between the orthologs.

Our functional studies demonstrate that BurK‐like enzymes are not restricted to malleicyprol producers. Enzymes inactivating reactive molecules such as the malleicyprols may function as protective agents that could grant a competitive advantage to the host as well as to potential mutualists. This concept has also been found in natural mutualistic interactions where “helper bacteria” protect fungi or microalgae by enzymatic toxin inactivation.^[^ ^35^, ^36^ ^]^ Given the unsatisfactory treatment options for melioidosis and glanders, we tested if a therapeutic microbe strategy might be envisaged to disarm the pathogens. To test this, we aimed to perform a protection assay with the model *C. elegans*, which is known to take up *E. coli* as a food source.

**Figure 5:**
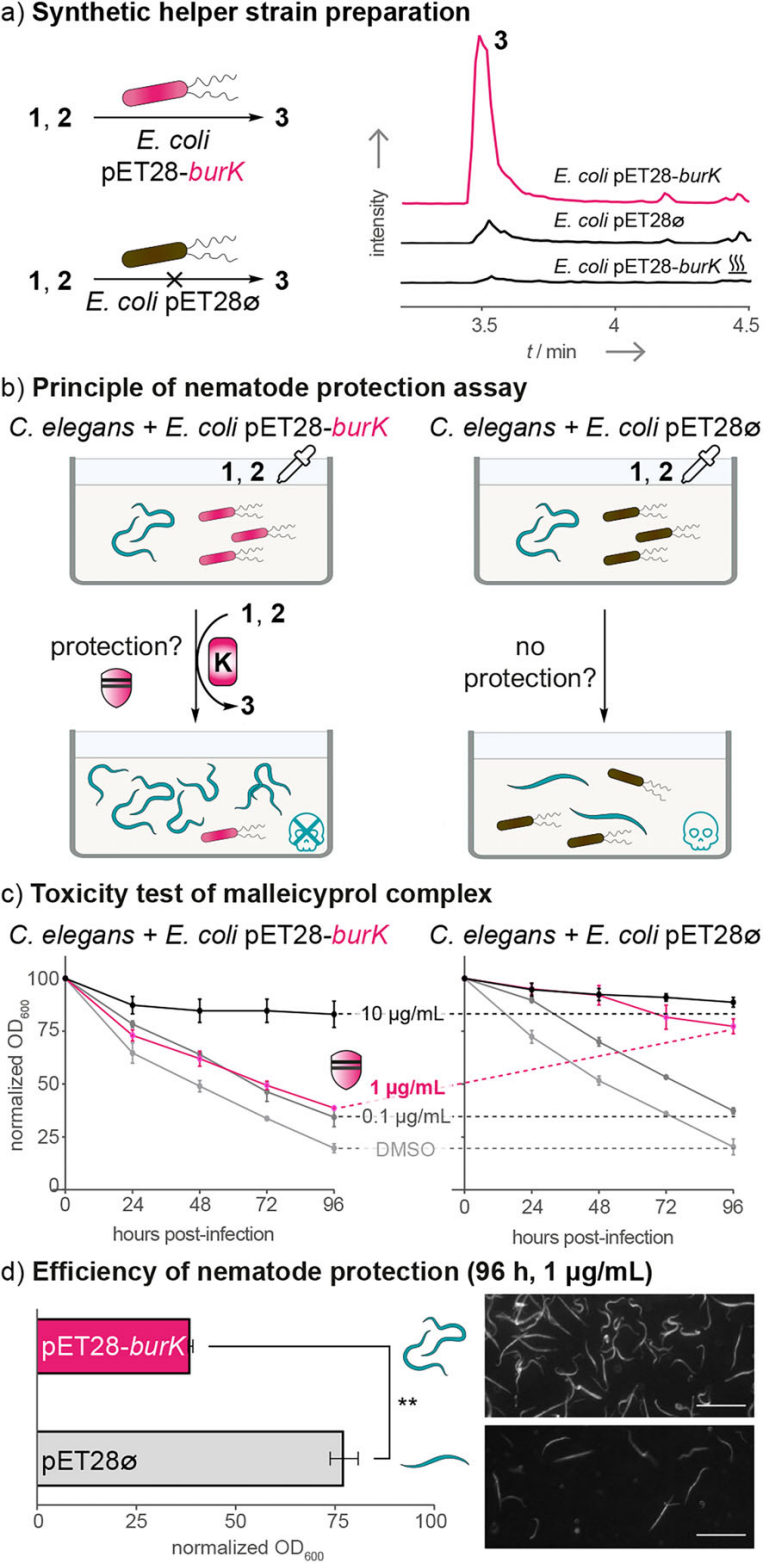
Nematode protection assay using the synthetic helper strain E. coli pET28‐*burK*. a) Heterologous expression of *burK* in a recombinant host (*E*. *coli* pET28‐*burK*) is sufficient to promote burkholderic acid formation (EIC: *m*/*z* 305.1758; [M − H]^−^) which indicates that the strain might be used to enzymatically inactivate toxins of the malleicyprol complex. b) Recombinantly produced BurK (from *E. coli* pET28‐*burK*) inactivates the malleicyprol complex (**1** and **2**), which should result in improved survival of C. elegans due to the conversion into burkholderic acid (**3**) by BurK. The empty‐plasmid strain (*E*. *coli* pET28∅) should serve as the negative control. c) Liquid feeding inhibition assay of *C. elegans* supplemented with various concentrations of **1** and **2** (0.1, 1, and 10 µg mL^−1^). Data points represent three independent replicated experiments (*n *= 3) OD_600_ ± 1 SEM. d) Efficiency of nematode protection from 1 µg mL^−1^ **1** and **2** after 96 h of co‐incubation. Data points are the same as depicted in panel B (*n* = 3 independent replicated experiments, OD_600_ ± one SEM). An unpaired *t*‐test with Welch's correction was performed (**, *p* < 0.01). Microscopic images of *C. elegans* after exposure to 1 µg mL^−1^ **1** and **2**. Nematodes were healthy and alive when feeding on *E. coli* pET28‐*burK* (OD_600_ = 40%, top panel). Incubation with *E. coli* pET28∅ showed impairment of the nematodes (OD_600_ = 78%, bottom panel). Scale bars: 500 µm. Representations of nematodes, pipettes, and skulls were adapted from illustrations obtained from Adobe Stock (see Supporting Information).

Therefore, we constructed an *E. coli* strain carrying a copy of *burK* as a synthetic helper bacterium. Specifically, we generated the expression plasmid pET28‐*burK*, heterologously produced BurK in *E. coli* Lemo21(DE3), and tested its potential to inactivate the toxin. As expected, induction of gene expression and addition of a malleicyprol‐containing extract from a Δ*burK* culture to the whole‐cell lysate leads to the formation of **3** (Figure 5a). Employing a heat‐inactivated cell lysate drastically reduced the formation of **3**. Small amounts of **3** were detected in the empty‐plasmid control suggesting that **3** may also be formed by another *E. coli* enzyme, but its formation is dramatically enhanced in the presence of BurK. Furthermore, addition of the radical‐trapping reagent **6** to the *burK*‐carrying strain generates **7** (Figure S14), consistent with the experiments carried out in *B. thailandensis*.

These results indicate that the pET28‐*burK*‐carrying strain could indeed be used as a therapeutic microbe that neutralizes malleicyprols. To test this, we performed a nematode protection assay using the *E. coli* pET28‐*burK* strain (Figure 5a). The *E. coli* strain harboring the empty plasmid (pET28∅) served as a negative control (Figure 5b). Both *E. coli* strains were co‐incubated with *C. elegans*, which was challenged with the malleicyprol complex at concentrations of 0.1 µg mL^−1^ (0.2 × IC_50_), 1 µg mL^−1^ (2 × IC_50_), and 10 µg mL^−1^ (20 × IC_50_)^[^ ^6^ ^]^ (Figure 5b). As described before, healthy nematodes feed on the bacteria (*E. coli*), leading to a reduction in the optical density (OD_600_). In contrast, if the nematodes are impaired, the OD_600_ remains close to the initial inoculation value (100%).

Exposure to 0.1 µg mL^−1^ of the malleicyprols (i.e., 0.2 × IC_50_) does not impair nematode survival as both *E. coli* strains (pET28‐*burK* and pET28∅) are readily consumed by the nematodes (Figure 5c). In contrast, treatment with 10 µg mL^−1^ of the toxin (i.e., 20  × IC_50_, a concentration that is assumed to exceed field concentrations) killed nearly all nematodes while analyzing both *E. coli* strains (Figure 5c). Importantly, when treating the nematodes with 1 µg mL^−1^ of the malleicyprols (i.e., 2 × IC_50_), we observed a normalized OD_600_ of 40% for *E. coli* pET28‐*burK*, whereas *E. coli* pET28∅ showed a normalized OD_600_ of 78% (Figure 5c). Thus, the malleicyprol‐inactivating *E. coli* pET28‐*burK* strain significantly reduces the toxicity of the malleicyprol complex (*p* < 0.01, Figure 5d) and promotes the survival of the nematodes. The finding that BurK exerts a protective effect against malleicyprols at concentrations in the range of the IC_50_ for *C. elegans* is intriguing from an ecological perspective as it relates to the concept of protective mutualism. Specifically, *E. coli* pET28‐*burK* functions as a helper bacterium that shields a partner (*C. elegans*) by enzymatically inactivating malleicyprol toxins, thereby enhancing the survival of the host. This principle mirrors natural biosystems such as mushroom‐ or microalga‐associated bacteria, which protect their hosts from antagonistic bacteria by cleaving their virulence factors.^[^ ^35^ ^]^

## Conclusion

This study provides insight into the inactivation of malleicyprol, a key virulence factor produced by pathogens of the *B. pseudomallei* group that cause severe disease in humans and animals. Using genetic manipulations, we show that the *burK* gene product generates the inactive constitutional isomer **3**. Our findings have implications for bioengineering, ecology, and medicine.

Through detailed biochemical and bioinformatic analysis, we identify BurK as a novel cytochrome *b* family oxidoreductase that employs a heme cofactor to facilitate cyclopropanol ring opening by a radical redox reaction. Although some enzymatic cleavages of cyclopropanes have been reported (e.g., colibactin cleavage,^[^ ^19^ ^]^ ethylene biosynthesis,^[^ ^37^ ^]^ or cycloeucalenol cleavage^[^ ^38^ ^]^), to date, no designated enzyme has been known that catalyzes the radical ring‐opening of a cyclopropanol moiety. BurK could serve as a scaffold for selective enzymatic β‐keto radical formation, a flourishing area in the field of organic synthesis where cyclopropanols are appreciated as versatile building blocks.^[^ ^30^, ^39^ ^]^

A nematode infection model further reveals that the BurK‐deficient strain is less virulent than BurK‐containing *Pbur*. Thus, production of the malleicyprol complex appears to be a double‐edged sword, requiring tight regulation of toxin levels to ensure full fitness and virulence in the host–pathogen context. This is in line with our discovery of orthologous enzymes in bacteria that do not possess the biosynthetic machinery for malleicyprol production. These BurK‐like enzymes may inactivate the reactive malleicyprol complex in ecological settings, thereby conferring advantages to their hosts or mutualistic partners. This concept of protective mutualism, in which helper bacteria shield their hosts by enzymatically inactivating toxins, has been observed across diverse ecological niches.^[^ ^35^, ^36^ ^]^

Finally, these findings have translational value. Enzymatic toxin cleavage, a strategy employed by helper bacteria in the environment,^[^ ^35^ ^]^ represents a promising “pathoblocker” approach using therapeutic microbes to combat bacterial infections.^[^ ^5^ ^]^ Specifically, results obtained from our nematode protection assay indicate that an avirulent BurK‐producing *E. coli* strain could mitigate *B. pseudomallei* toxicity by neutralizing the malleicyprol complex. This strategy serves as a foundation for developing antivirulence therapeutics against *B. pseudomallei* infections. Beyond potential clinical applications, this approach could also help to sanitize *B. pseudomallei*‐contaminated environments. Related strategies are already used for environmental detoxification of pollutants (e.g., microcystins^[^ ^40^ ^]^) or in the food industry to inactivate food contaminants (e.g., aflatoxin^[^ ^41^ ^]^ and deoxynivalenol^[^ ^42^ ^]^). In illuminating the enzymatic toxin inactivation by BurK as a novel mediator modification,^[^ ^43^ ^]^ we lay the foundation for the development of a biocatalytic pathoblocker with potential applications in both therapeutic intervention and environmental decontamination of pathogens belonging to the *B. pseudomallei* group.

## Supporting Information

The data that support the findings of this study are available in the Supporting Information of this article.

## Author Contributions

Jonas Fiedler and Christian Hertweck designed research. Jonas Fiedler, Ingrid Richter, Katharina Dornblut, and Alicia Scharf performed research. Jonas Fiedler, Ingrid Richter, Katharina Dornblut, and Alicia Scharf analyzed data. Jonas Fiedler, Ingrid Richter, and Christian Hertweck wrote the paper.

## Conflict of Interests

The authors declare no conflict of interest.

## Supporting information



Supporting Information

## Data Availability

The data that support the findings of this study are available in the Supporting Information of this article.
