# Supplementary material for: Inactivation of the Burkholderia Toxin Malleicyprol by Enzymatic Cyclopropanol Ring Opening
Source: Angew Chem Int Ed Engl. 2025 Nov 10;65(2):e21105. doi: 10.1002/anie.202521105 (PMC12790350; doi:10.1002/anie.202521105)
Supplement: Supplementary file 1 — Supporting Information [file ANIE-65-e21105-s001.pdf]

SUPPORTING INFORMATION

Table of contents

General methods 1
Molecular biology 1
Construction of pJET-*burK*-KO 1
Preparation of *B. thailandensis Pbur*Δ*burK* 1
Construction of pSCrhaB2-*burK* 2
Preparation of *B. thailandensis Pbur*Δ*burK* pSCrhaB2-*burK* 2
Site-directed mutagenesis of pSCrhaB2-*burK* 2
Complementation of *B. thailandensis Pbur*Δ*burK* with point mutation variants 2
Analysis of point mutation variants 2
Radical trapping in *B. thailandensis* strains 3
Generation of pET28-*burK* 3
Recombinant production of BurK 3
In vitro formation of burkholderic acid using recombinant BurK 3
Radical trapping in *E. coli* strains 3
Construction of pSCrhaB2 plasmids containing *burK* homologs and genetic complementation 4
Analysis of *burK* variants 4
Bioinformatic analysis 4
General analysis of metabolites 4
Nematode infection assay 5
Nematode protection assay 5
Growth comparison of *Pbur* and Δ*burK* 5
Agar diffusion test 5
Analytical procedures 6
Image credits 6
Amino acid sequences 7
Supplementary figures 8
Supplementary tables 17
References 24
Author contributions 25

List of supplementary figures

Figure S1. Biosynthesis of malleicyprol. 8

Figure S2. Genetic inactivation of *burK*. 9

Figure S3. Genetic complementation of *Pbur*Δ*burK*. 9

Figure S4. Growth comparison of *Pbur* and Δ*burK* and agar diffusion test. 10

Figure S5. Distribution of *burK* in putative malleicyprol producers. 10

Figure S6. Microscopic images of nematodes feeding on different *B. thailandensis* strains. 11

Figure S7. Sequence analyses of BurK. 12

Figure S8. Comparison of BurK and CypB. 13

Figure S9. Alternative reaction pathway. 14

Figure S10. Protein–ligand co-folding model. 14

Figure S11. MS^2 analysis of PBN-adduct. 15

Figure S12. Phylogeny of BurK and related cytochrome b enzymes. 15

Figure S13. Sequence comparison of BurK-like enzymes. 16

Figure S14. Radical-trapping experiment using *E. coli* strains. 16

List of supplementary tables

Table S1. Transcriptome analysis. 17
Table S2. Transposon analysis. 17
Table S3. Analyzed BurK-like proteins. 17
Table S4. Protein accession numbers of proteins used for the phylogeny. 18
Table S5. Strains used in this study. 21
Table S6. Plasmids used in this study. 22
Table S7. Oligonucleotide primers used in this study. 23

Experimental Procedures

General methods

Media components were obtained from Carl Roth (Karlsruhe, Germany).
Oligonucleotide primers were supplied from Eurofins Genomics (Ebersberg, Germany). Plasmid sequencing was carried out by Genewiz (Leipzig, Germany).
Glycerol stocks were prepared by mixing 500 µL of an overnight culture with 500 µL 40 % glycerol (in water; *v*/*v*) and the glycerol stocks were stored at −70 °C. Optical density was measured at 600 nm (OD_{600}) using a biophotometer from Eppendorf (Hamburg, Germany). Bacterial cultures were agitated in an orbital shaker and the shaking velocity is reported in revolutions per minute (rpm). ChemBioDraw 12.0 was used to visualize all compounds and to calculate *m*/*z* values.

Molecular biology

Transformations, DNA manipulations and agarose gels were carried out with established methods.[1] Plasmid DNA was purified with a Monarch® Plasmid Miniprep kit from New England Biolabs (NEB) (Frankfurt am Main, Germany). The Monarch® PCR & DNA Cleanup kit from NEB was used for DNA purification from restriction endonuclease reactions, ligation reactions, and homology reactions. The Monarch® DNA Gel Extraction kit was used for DNA purification from agarose gels after PCRs or plasmid digestions. The NEBuilder kit (NEB) was used for DNA assembly when indicated. All kits were used according to the manufacturers' instructions. Restriction endonucleases from NEB were used as described form the manufacturer. The T4 DNA ligase (NEB) was used for ligation reactions. Genomic DNA isolation was carried out with the Wizard® Genomic DNA Purification Kit (Promega, Madison, USA) according to the manual.
Plasmid pJET1.2 from Thermo Fisher Scientific (Waltham, USA) was used for homologous recombination. pET28a(+) from Merck Millipore (Darmstadt, Germany) was used for enzyme production. pSCrhabB2 was used for genetic complementation.[2]

Construction of pJET-*burK*-KO

Genomic DNA of *B. thailandensis* E264 served as template for PCR amplification of two gene fragments containing gene fragments of *burJ* and *burL* (both 1,000 bp) (primer pairs: and *burK*_KO1_F/*burK*_KO1_R and *burK*_KO2_F/*burK*_KO2_R). PCRs were carried out using Q5 polymerase (NEB). Additionally, the kanamycin resistance cassette (Kan^R) was PCR-amplified from the plasmid pET28 (primer pair: *kan*_F/*kan*_R). All amplicons were gel-purified and assembled with a pJET1.2 vector backbone using the NEBuilder kit generating pJET-*burK*-KO.

Preparation of *B. thailandensis Pbur*Δ*burK*

A freshly grown colony of *B. thailandensis* E264 *Pbur* was used to inoculate a preculture in lysogeny broth[3] (LB) medium (20 mL supplemented with tetracycline 45 µg/mL, 30 °C and 130 rpm). Cultured cells were used to inoculate a main culture (200 mL, LB-medium) to an OD_{600} = 0.1. Cells grew until the OD_{600} reached 0.6. The culture was then transferred to four 50 mL tubes, and they were centrifuged (5 min, 6,000 × *g*, room temperature). The precipitated cells were then washed with 20 mL sucrose (300 mM) and the centrifugation step was repeated. The washing step was repeated twice and after the last washing step, the supernatant was removed, and four 100 µL aliquots were prepared by addition of 400 µL sucrose (300 mM). The plasmid pJET_*burK*_KO (40 ng/µL) was heated for 5 min at 98 °C, shortly cooled to room temperature, and then transferred to the aliquots using different amounts of plasmid solution (1 µL, 5 µL, 10 µL, or 20 µL). Each solution was then transferred to electroporation cuvettes and subjected to electroporation (2,250 mV). Transformed cells were subsequently grown in LB medium (2 mL) for 5 h (30 °C, 150 rpm) and were then spread on LB agar plates supplemented with tetracycline (45 µg/mL) and kanamycin (150 µg/mL). Some colonies were observed three days later. Single colonies were used to inoculate 2 mL LB medium supplemented with tetracycline and kanamycin (same concentrations as stated before). After overnight growth, the cells were used for gDNA extraction and the extracted gDNA served as template for PCR gene amplification using the primer pair *burK*_KO_screening_fw/ *burK*_KO_screening_rv using OneTaq master mix (NEB). Positive clones were used for glycerol stock preparation.

Construction of pSCrhaB2-*burK*

The plasmid pSCrhaB2 was treated with the restriction endonucleases *Nde*I and *Bam*HI. The *burK* gene was amplified from genomic DNA of *B. thailandensis* E264 using the primer pair *burK*_compl_F/*burK*_compl_R with Q5 polymerase and the generated amplicon was purified by gel extraction. The purified fragment was treated with *Nde*I and *Bam*HI, purified by column-purification and ligated with the pSCrhaB2 plasmid backbone. The prepared pSCrhaB2-*burK* plasmid was verified by sequencing.

Preparation of *B. thailandensis Pbur*Δ*burK* pSCrhaB2-*burK*

Electrocompetent *B. thailandensis Pbur*Δ*burK* cells were prepared according to a published method.[4] A 20 mL culture was grown in LB medium (containing 45 µg/mL tetracycline and 150 µg/mL kanamycin) at 30 °C overnight. The culture (4 × 1.5 mL aliquots) was centrifuged (16,000 × *g*, room temperature, 1 min) and the supernatant was discarded. The cells were resuspended in 1 mL of 300 mM sucrose and centrifuged again under the same conditions. This washing step was repeated twice and the aliquots were combined. The final cell pellet was suspended in 100 µL 300 mM sucrose and subjected to electroporation with 300 ng of plasmid pSCrhaB2-*burK* at 2,250 mV.
After electroporation, the cells were recovered in 2 mL LB medium and incubated at 30 °C with shaking (150 rpm) for 4 hours. The culture was then spread on LB agar plates containing tetracycline (45 µg/mL), kanamycin (150 µg/mL), and trimethoprim (100 µg/mL). Colonies appeared after three days. A single colony was used to inoculate 2 mL LB medium supplemented with the same antibiotics and grown overnight. Plasmid DNA was extracted from this culture, sequenced, and used as a template for PCR confirmation using primers pSCrhaB2_f and pSCrhaB2_r with Q5 DNA polymerase.

Site-directed mutagenesis of pSCrhaB2-*burK*

The point mutations were constructed using the Q5 site-Directed Mutagenesis Kit (NEB) following the manufactures instructions and employing the mutagenesis primers (Table S7). However, the stand-alone Q5 high-Fidelity DNA polymerase (NEB) with the GC enhancer (NEB) was used instead of the kit component Q5 Hot Start High-Fidelity 2X Master Mix (NEB).
After transformation, the culture was spread on LB agar plates containing trimethoprim (100 µg/mL). After incubation at 37 °C overnight, colonies were visible the next day. Next, individual single colonies were grown; the plasmids were purified and sent for whole-plasmid sequencing to verify the presence of the correct mutation and the plasmid backbone.

Complementation of *B. thailandensis Pbur*Δ*burK* with point mutation variants

The same electroporation protocol was used as described for the preparation of *B. thailandensis Pbur*Δ*burK* pSCrhaB2-*burK* using the respective plasmids. Plasmid DNA was extracted from the generated strains, sequenced, and used as a template for PCR confirmation using primers pSCrhaB2_f and pSCrhaB2_r with Q5 DNA polymerase.

Analysis of point mutation variants

A glycerol stock of each strain was spread onto LB agar containing tetracycline (45 µg/mL), kanamycin (150 µg/mL) and trimethoprim (100 µg/mL). After three days, liquid LB medium containing the same antibiotics (2 mL, in 10 mL culture tubes) was inoculated with a single colony of each strain (in total three for each strain). The culture was incubated for 16 h at 150 rpm and 30 °C. Subsequently, 100 mL flasks were prepared containing MM9 medium[5] (20 mL) supplemented with the mentioned antibiotics and 0.02 % rhamnose. They were inoculated with 200 µL of each pre-culture and grown for 16 h at 150 rpm and 30 °C. The resulting cultures (1 mL each) were extracted with ethyl acetate (1 mL). The organic phases were concentrated under reduced pressure, dissolved in 40 µL methanol and subjected to HPLC-HRMS analysis using method B.
Generated data files (RAW format) were converted to mzML format using msConvert (version 3.0).[6] Then, they were analyzed in mzMine (version 4.5.37) using the batch mode.[7] Mass detection was employed in the negative polarity mode for the complete chromatography run and subsequently the chromatogram builder algorithm was used (default settings, retention time filter: 8.45–8.90 min). A feature list row filter (*m*/*z* 305.1750–305.1770) was then applied to identify signals originating from burkholderic acid. The peak area was then exported to Microsoft Excel (Version 2503) and the obtained data was processed and visualized in GraphPad Prism (version 10.4.0). The determined mean peak area of extracts of the *B. thailandensis Pbur*Δ*burK* pSCrhaB2 cultures was used as blank and was subtracted from each obtained value.

Radical trapping in *B. thailandensis* strains

A glycerol stock of *Pbur* or *Pbur*Δ*burK* was spread onto LB agar either containing tetracycline (45 µg/mL) or tetracycline (45 µg/mL) and kanamycin (150 µg/mL). After three days, liquid LB medium containing the same antibiotics (20 mL, in 100 mL flask) was inoculated with a single colony of each strain. The culture was incubated for 16 h at 150 rpm and 30 °C. Subsequently, 100 mL flasks were prepared containing MM9 medium (20 mL) supplemented with the mentioned antibiotics. They were inoculated with 200 µL of each pre-culture and grown for 5 h at 150 rpm and 30 °C. Then, *N*-tert-butyl-α-phenylnitrone (supplied from TCI; Zwijndrecht, Belgium) was added to the cultures (final concentration: 1 mM, stock concentration: 1 M; dissolved in DMSO) and incubation continued overnight.
The resulting cultures (1 mL each) were extracted with ethyl acetate (1 mL). The organic phases were concentrated under reduced pressure, the extracts dissolved in 40 µL methanol and subjected to HPLC-HRMS analysis using method B.

Generation of pET28-*burK*

The *burK* gene was amplified from genomic DNA of *B. thailandensis* E264 using the primer pair *burK*_recom_F_*Nco*I and *burK*_recom_R_*Xho*I with Q5 DNA polymerase. The resulting PCR product was purified by gel extraction and treated with *Nco*I and *Xho*I after which the fragment was column-purified. The pET28 plasmid was also incubated with *Nco*I and *Xho*I and purified by gel-extraction. The plasmid was then ligated with the *burK* fragment to generate pET28-*burK*.

Recombinant production of BurK

E. coli Lemo BL21 (DE3) was transformed with the plasmid pET28-*burK* or pET28. LB-medium containing kanamycin (50 µg/mL) was inoculated with a freshly grown colony of the respective strain and incubated overnight (37 °C, 150 rpm). The obtained culture (200 µL) was used to inoculate 20 mL LB medium supplied with kanamycin (50 µg/mL) in a 100 mL baffled Erlenmeyer flask. The cells were grown at 37 °C until the OD_{600} reached 0.6–1 (approximately 3 h). Then, isopropyl β-D-1-thiogalactopyranoside (IPTG) was added to a final concentration of 100 µM. The fermentation was continued overnight (21 °C, 150 rpm). The obtained cultures were centrifuged (20 min, 5,000 × *g*), and the resulting cells were washed with phosphate buffer (50 mL; 150 mM phosphate buffer pH 7.0, containing 125 mM NaCl). The centrifugation step was repeated, the supernatant was discarded, and the cells were then stored at −20 °C.

***In vitro* formation of burkholderic acid using recombinant BurK**

Frozen cells (*E. coli* Lemo BL21 (DE3) pET28-*burK* or empty-control strain) were suspended in 1 mL of phosphate buffer per gram of pellet and lysed by sonication (3 × 2 min, 70 % power). The resulting lysate (800 µL) was kept on ice, and 10 µL of a *Pbur*Δ*burK* extract (5 mg · mL^{-1}) was added. Samples were then incubated overnight at 30 °C with shaking (400 rpm, Eppendorf ThermoMixer® C), followed by extraction with 800 µL of ethyl acetate. The organic phase (750 µL) was separated and concentrated using a vacuum centrifuge (Eppendorf Concentrator plus). For HPLC-HRMS analysis, the dried residue was dissolved in 40 µL of methanol and analyzed using method A. A heat-inactivated lysate (10 min, 98 °C) and the empty-plasmid culture served as negative controls.

Radical trapping in *E. coli* strains

The *E. coli* strains (*E. coli* Lemo BL21 (DE3) pET28-*burK* or *E. coli* Lemo BL21 (DE3) pET28; glycerol stocks) were spread onto LB agar plates containing kanamycin (50 µg/mL) and incubated at 37 °C. After incubation (16 hours), a colony was picked from each plate and used to inoculate 20 mL LB medium containing kanamycin (50 µg/mL) in a 100 mL baffled flask. The cultures were then incubated for 16 h and 150 rpm at 37 °C. From these pre-cultures, main culture were prepared (75 mL LB medium; containing kanamycin, 50 µg/mL; in a 300 mL baffled flask) which were incubated at 37 °C and 180 rpm. After 3 h, IPTG was added to a final concentration of 1 mM. The fermentation was continued overnight (21 °C, 180 rpm) and then used for radical trapping experiments.
The cultures were transferred to centrifugation tubes and centrifuged (10 min, 4 °C, 4,000 × *g*), the supernatant was discarded, and the resulting cells were washed with phosphate buffer (20 mL; 150 mM phosphate buffer pH 7.0, containing 125 mM NaCl). The centrifugation step was repeated (10 min, 4 °C, 4,000 × *g*) and the supernatant discarded. The cells were then suspended in phosphate buffer (1 mg/mL). The suspension (350 µL) was then treated with an extract (18.4 mg/mL, 12.5 µL) derived from Δ*burK* and **6** (3.5 µL, final concentration: 1 mM, stock concentration: 100 mM; dissolved in DMSO) if indicated. The mixture was then incubated at 30 °C with shaking (800 rpm, Eppendorf ThermoMixer® C) for 23 hours, followed by extraction with 400 µL of ethyl acetate. The organic phase (300 µL) was separated and concentrated using a vacuum centrifuge (Eppendorf Concentrator plus). For HPLC-HRMS analysis, the dried residue was dissolved in 40 µL of methanol and analyzed using method B.

Construction of pSCrhaB2 plasmids containing *burK* homologs and genetic complementation

Synthetic pET28 plasmids containing *burK* orthologs were ordered from Twist Bioscience (San Francisco, USA). The respective inserts were PCR-amplified using the indicated primers (Table S7) and cloned into pSCrhaB2 plasmids (*Nde*I/*Bam*HI) using the NEBuilder kit by homologous recombination.
The same electroporation protocol was used as described for the preparation of *B. thailandensis Pbur*Δ*burK* pSCrhaB2-*burK* using the respective plasmids to generate cross-complemented strains.

Analysis of *burK* variants

A glycerol stock of each strain was spread onto LB agar containing tetracycline (45 µg/mL), kanamycin (150 µg/mL) and trimethoprim (100 µg/mL). After three days, liquid LB medium containing the same antibiotics (2 mL, in 10 mL culture tubes) was inoculated with a single colony of each strain (three colonies for each strain). The culture was incubated for 16 h at 150 rpm and 30 °C. Subsequently, 100 mL flasks were prepared containing MM9 medium (20 mL) supplemented with the mentioned antibiotics and 0.02 % rhamnose. They were inoculated with 200 µL of each pre-culture and grown for 16 h at 150 rpm and 30 °C. The resulting cultures (1 mL each) were extracted with ethyl acetate (1 mL). The organic phases were concentrated under reduced pressure, and the obtained extracts were dissolved in 40 µL methanol and subjected to HPLC-HRMS analysis using method B.
The same analysis workflow was used as described for the analysis of BurK point mutants.

Bioinformatic analysis

The BurK amino acid primary sequence was analyzed using different programs. The ProtParam program from the ExPASy webserver was used to calculate the isoelectric point (pI) and the amino acid composition.[8] The Phyre^2 server (version 2.2) was used in the 'normal' modeling mode to predict transmembrane helices.[9] The TMHMM server was also used to analyze transmembrane helices.[10] The HHPred server and the Dali server were used to detect structural homologs with default parameters.[11]

Gene clusters used to identify the presence of *burK* were retrieved from the EFI-GNT webserver using default parameters, with BurG as the query. If a gene cluster was present in many subspecies, only one representative cluster was shown for that species.

The AlphaFold Server (https://golgi.sandbox.google.com/)[12] was used to predict the tertiary structure of BurK and BurK-like enzymes. When indicated, two Heme *b* cofactors were integrated into the models. Structural superimpositions were performed using ChimeraX (version 1.7)[13] and the matchmaker algorithm with default parameters.
The protein–ligand co-folding model was generated by Boltz-2,[14] using the Rowan Scientific environment (https://www.rowansci.com, accessed 2025-07-15).

Phylogenetic analysis was performed with Geneious Prime 2020.2.4 (https://www.geneious.com). Protein sequences (Table S4) were aligned by using the MAFFT algorithm with default parameters. The alignment was subsequently used for phylogenetic tree generation with the FastTree program with default parameters. CybB-like[15] and FcrB-like enzymes[16] were obtained from previously published lists. HemJ-like enzymes[17] were retrieved from the NCBI protein database using HemJ as a BLAST query, followed by a second query using a HemJ ortholog from *B. thailandensis*. CtaA-like[18] and CypP-like enzymes[19] were identified using the respective protein sequences as BLAST queries, either against the entire database or restricted to the *Burkholderia* group; in both cases, the top 10 hits were used for downstream analyses. CtaA-like proteins (consisting of two four-helical bundles) were trimmed to helix 5 to 8 (the helices that coordinate the heme cofactor). BurK-like enzymes from the SSN were also included in the phylogeny.

BurK-like proteins were aligned by using the MAFFT algorithm with default parameters in Geneious Prime 2020.2.4. The generated alignment was forwarded to the ESPript server[20] which was employed with default parameters.

General analysis of metabolites

MM9 medium (20 mL) was inoculated with a freshly grown colony of *B. thailandensis Pbur*, *B. thailandensis Pbur*Δ*burK*, *B. thailandensis Pbur*Δ*burK* pSCrhaB2-*burK*, or *Pbur*Δ*burK* pSCrhaB2 respectively, and incubated overnight with agitation (30 °C and 150 rpm). The overnight cultured cells were used to inoculate 100 mL MM9 medium to an OD_{600} of 0.1 in a 300 mL baffled Erlenmeyer flask. Sterile-filtered L(+)-rhamnose (20 % *w*/*v* in water; 100 µL, final concentration of 0.02 %) was added to the culture of *B. thailandensis Pbur*Δ*burK* pSCrhaB2-*burK* and *B. thailandensis Pbur*Δ*burK* pSCrhaB2. The cultures were then incubated for 16 h at 30 °C with orbital shaking at 150 rpm.
The resulting cultures were extracted with ethyl acetate (2 × 100 mL). The combined organic phases were dried with Na_2SO_4, filtered, and concentrated under reduced pressure. The obtained extracts were dissolved in methanol to a concentration of 1 mg/mL, filtered through a 0.2 µm PTFE ROTILABO syringe filter, subjected to HPLC-HRMS analysis and analyzed using method A.

Nematode infection assay

Wild-type *Caenorhabditis elegans* strain N2 (var. Bristol) was obtained from the *C. elegans* Genetics Center (CGC, University of Minnesota) and maintained on nematode growth medium (NGM) seeded with *E. coli* OP50 as a food source.[21] For feeding inhibition assays, nematode suspensions were prepared as previously described.[22] Liquid bacterial cultures (*Pbur*, Δ*burK*, *ΔburG*, *ΔburK*-comp, and *ΔburKØ*) were grown in LB medium at 37 °C and 150 rpm overnight. Cells were harvested by centrifugation at 8,000 rpm for 5 min and resuspended in K-medium (3.1 g/L NaCl, 2.4 g/L KCl) and the OD_{600} was adjusted to 1.2. Individual wells of six-well cell culture plates (Costar, Corning) were seeded with 1.8 mL of bacterial suspension (OD_{600}: 1.2) and 200 µL of nematode suspension. To assess the natural viability of bacterial cells, control wells without nematodes were included in each assay. All plates were incubated at 20 °C with shaking at 50 rpm for 5 days, and OD_{600} was measured every 24 h. The number of viable nematodes in the suspension was inferred from the corresponding bacterial cell density. OD_{600} values obtained from wells with nematodes were normalized against values from wells without nematodes. Normalized mean OD_{600} values from three independent experiments ($n = 3$ biological replicates) were plotted as a percentage of the initial OD_{600} ± standard error of the mean (SEM).

Nematode protection assay

The synthetic helper strain (*E. coli* Lemo BL21 (DE3) pET28-*burK*) and the empty-plasmid control strain were spread onto LB agar plates containing kanamycin (50 µg/mL). After two days, a colony was picked from the plate and used to inoculate 20 mL LB medium containing kanamycin (50 µg/mL) in a 100 mL baffled flask. The cultures were then incubated for 16 h and 150 rpm at 37 °C. From these pre-cultures, a main cultures were prepared (75 mL LB medium; containing kanamycin, 50 µg/mL; in a 300 mL baffled flask), which were incubated at 37 °C and 180 rpm. After 3 h, IPTG was added to a final concentration of 1 mM. The fermentation was continued overnight (21 °C, 180 rpm) an then used in the nematode protection assay.

For potency assessment, 200 µL of nematode suspension was added to 1.76 mL of bacterial cell suspension (*E. coli* pET28-*burK* and *E. coli* pET28*Ø*) at OD_{600} 1.2. The malleicyprol complex (**1** and **2**) was dissolved in DMSO and added (40 mL) to the wells with a final concentration of 0.1 µg/mL, 1 µg/mL, or 10 µg/mL (three technical replicates per concentration). The OD_{600} values were measured as described above. Normalized mean OD_{600} values from three independent experiments ($n = 3$ biological replicates) were plotted as a percentage of the initial OD_{600} ± standard error of the mean (SEM). Statistical analysis was performed in GraphPad Prism 6.03. An unpaired *t* test with Welch's correction was used to study the relationship between nematodes feeding on *E. coli* pET28-*burK* and *E. coli* pET28*Ø*. *P* values of < 0.05 were considered statistically significant.

Growth comparison of *Pbur* and Δ*burK*

The growth of *Pbur*, Δ*burK* and Δ*burM* was analyzed using a Varioskan LUX multimode microplate reader (Thermo Fisher Scientific). A 20 mL culture was grown in LB medium (containing appropriate antibiotics) at 30 °C overnight. The culture was then used to inoculate 5 mL LB medium to an $OD_{600} = 0.01$. The inoculated culture (200 µL for each well) was then placed into ten wells of a 96-well plate. Surrounding wells contained either medium or other test strains (200 µL). The 96-well plate was then inserted into the Varioskan LUX microplate reader with the lid on. The optical density (absorbance at 600 nm) was then measured every 15 min overnight, with incubation at 30 °C and continuous low-force shaking at 60 rpm in the kinetic measurement mode.

Agar diffusion test

A 20 mL culture of *PburΔburK* was grown overnight in LB medium (supplemented with appropriate antibiotics) at 30 °C. Liquid LB agar (1.5 %, 500 mL) was kept in a 45 °C water bath, and a 20 mL aliquot was inoculated with the overnight culture to an OD_{600} of 0.01. The inoculated agar was poured into a sterile petri dish and allowed to solidify at room temperature for 5 minutes. Once solidified, a sterile paper disc (10 mm) was placed on the surface. Then, 20 µL of bis-malleicyprol (dissolved in methanol, 1,500 µg/mL) was applied to the center of the disc. The agar plate was incubated overnight at 30 °C and subsequently examined for bacterial growth inhibition, which would be indicated by a clear halo zone around the disc.

Analytical procedures

HPLC-HRMS data were obtained on an UltiMate 3000 HPLC (Thermo Fisher Scientific), coupled to a QExactive HF-X Hybrid Quadrupole-Orbitrap (Thermo Fisher Scientific) using electrospray ionization (ESI). $HRMS^2$ measurements were carried out in Parallel Reaction Monitoring (PRM) mode. All Data are presented as mass-to-charge ratio (*m/z*).
Method A: General, fast method for analysis of extracts. Nucleodur HTec C_{18} (100 × 2 mm, 1.8 µm, Macherey-Nagel), elution gradient: [solvent A: H_2O + 0.1 % formic acid, solvent B: acetonitrile + 0.1 % formic acid, 5 % to 98 % B in 7 min, 98 % B for 3 min,100 % B to 5 % B in 0.001 min, 5 % B for 1.5 min; flow rate: 0.4 mL/min, injection volume: 2 µL].

HPLC-HRMS was also performed on an UltiMate 3000 HPLC (Thermo Fisher Scientific), coupled to a QExactive Hybrid-Quadrupole-Orbitrap mass spectrometer (Thermo Fisher Scientific) using ESI. $HRMS^2$ measurements were carried out in PRM mode.
Method B: Analysis of production levels of burkholderic acid, due to better peak shape and subsequent peak integration. Accucore C_{18} column (2.1 × 100 mm, 2.6 µm, Thermo Fisher), elution gradient [solvent A: H_2O + 0.1 % formic acid, solvent B: acetonitrile + 0.1 % formic acid, 5 % B for 1 min, 5 % to 98 % B over 10 min, hold 98 % B for 12 min; flowrate 0.2 mL/min, injection volume: 2 µL].

Metabolomics analysis was carried out using the software Compound Discoverer (v3.3) from Thermo Fisher Scientific. The quick-metabolomics workflow ("Untargeted Metabolomics Quick Detection Unknowns No ID") was used with minor adjustments (polarity: negative; quality threshold filter: 5; intensity threshold: 10^6, baseline removed). Both strains (*Pbur*, *Pbur*Δ*burK*) were compared to each other by differential analysis. Two cultures of each strain were prepared on two different days as described in the section "General metabolite analysis" and analyzed with HPLC-HRMS method A.

Image credits

Some representations in Figure 5 and the graphical table of contents were obtained or adopted from Adobe Stock (stock.adobe.com, business design plan).
Pipette from "SVIATOSLAV" (Stock-ID: #438617348), nematodes from "megis" (Stock-ID: #1489949731), skull from "VektorDesign" (Stock-ID: #1046664318), magnifying glass from "popcornarts" (Stock-ID: #486648695), monitor from "fendy" (Stock-ID: #303750723), lake and forest representation from "AI Art Factory" (Stock-ID: #1525843962), lung from "Parbat" #943658159, shield from "warmworld" (Stock-ID: #354553493).

Amino acid sequences

The amino acid residues that were mutated are highlighted in red. The artificial amino acids of the His_6-tag of the recombinant protein are separated from amino acids of native BurK by a hyphen. A non-native alanine residue in BurK-*C*-His_6 is highlighted in bold. This residue was added to keep the correct reading frame.

BurK:

MKLVLFLHLIFVAAWMSCVIVEGIFEHAIDRSPEQRAFISKLHWSTDKYVEIPAFTIVLITGAILLAHRAPTPLLLTKVAFGTLAIALNAVCVWIVIR
RTRYAARNDYAAWERIDRLQHKLGGVVAISMLVALGIGGYLFAGG

BurK-H8A:

MKLVLFLALIFVAAWMSCVIVEGIFEHAIDRSPEQRAFISKLHWSTDKYVEIPAFTIVLITGAILLAHRAPTPLLLTKVAFGTLAIALNAVCVWIVIR
RTRYAARNDYAAWERIDRLQHKLGGVVAISMLVALGIGGYLFAGG

BurK-H43A:

MKLVLFLHLIFVAAWMSCVIVEGIFEHAIDRSPEQRAFISKLAWSTDKYVEIPAFTIVLITGAILLAHRAPTPLLLTKVAFGTLAIALNAVCVWIVIR
RTRYAARNDYAAWERIDRLQHKLGGVVAISMLVALGIGGYLFAGG

BurK-K78A:

MKLVLFLHLIFVAAWMSCVIVEGIFEHAIDRSPEQRAFISKLHWSTDKYVEIPAFTIVLITGAILLAHRAPTPLLLTAVAFGTLAIALNAVCVWIVIR
RTRYAARNDYAAWERIDRLQHKLGGVVAISMLVALGIGGYLFAGG

BurK-H119A:

MKLVLFLHLIFVAAWMSCVIVEGIFEHAIDRSPEQRAFISKLHWSTDKYVEIPAFTIVLITGAILLAHRAPTPLLLTKVAFGTLAIALNAVCVWIVIR
RTRYAARNDYAAWERIDRLQAKLGGVVAISMLVALGIGGYLFAGG

BurK-Pse:

MVTLLLQLHLFAVAFWLGVVAVEFLLERSRSQSRGQGFAVADLHGRIDLLFETPAFVTVLFSGLLLLDAERFSGVYAVKVVAGMVAVLGNVLCVIPVL
LRRTAAGAGDLPAVIRFSRQIDVISLLAIPAGLLALLLGTYLVVTR

BurK-Alc:

MANSAIVLKLHLFGVAFWLGVVGVEFLLERRRALSREHGYLVARLHERIDLCLEMPAFLLVLVTGLLLLDVEQLSGVYLLKVLAGLVAVSGNLLCLWP
VMCRKLAADRGDLPRVIHYSNVIDRISVVAIPAGLFAFACGVWLVISR

BurK-Rho:

MAPSYLLPLHLVFVGVWLGCVLTEALFERALLGQGRAFELVLVALHKKVDLLIEIPAFVGVLVTGALMLHAAPPSNLLHLKAGLGVLAIAANAYCVWL
VFRRGDAAQAGDWERFERIDHAQHTWGAVVLVGILAALGIGLYLFSHG

BurK-Tha:

MANIFLFIHLIALGIWAGCVLTEVMLELVLEKLPPESSNLARLHAMIDRFVEIPAILLVAITGGMLLQSAYWDTLLQVKVGLGVTAVFLNTVAAFTVQ
RRYQSLLSGNEQGYDFWNLWHERTGIGCVLSITGAIVVGGYRLVGAG

BurK-*C*-His_6:

M**A**KLVLFLHLIFVAAWMSCVIVEGIFEHAIDRSPEQRAFISKLHWSTDKYVEIPAFTIVLITGAILLAHRAPTPLLLTKVAFGTLAIALNAVCVWIVI
RRTRYAARNDYAAWERIDRLQHKLGGVVAISMLVALGIGGYLFAGG-
LEHHHHHH

Supplementary figures

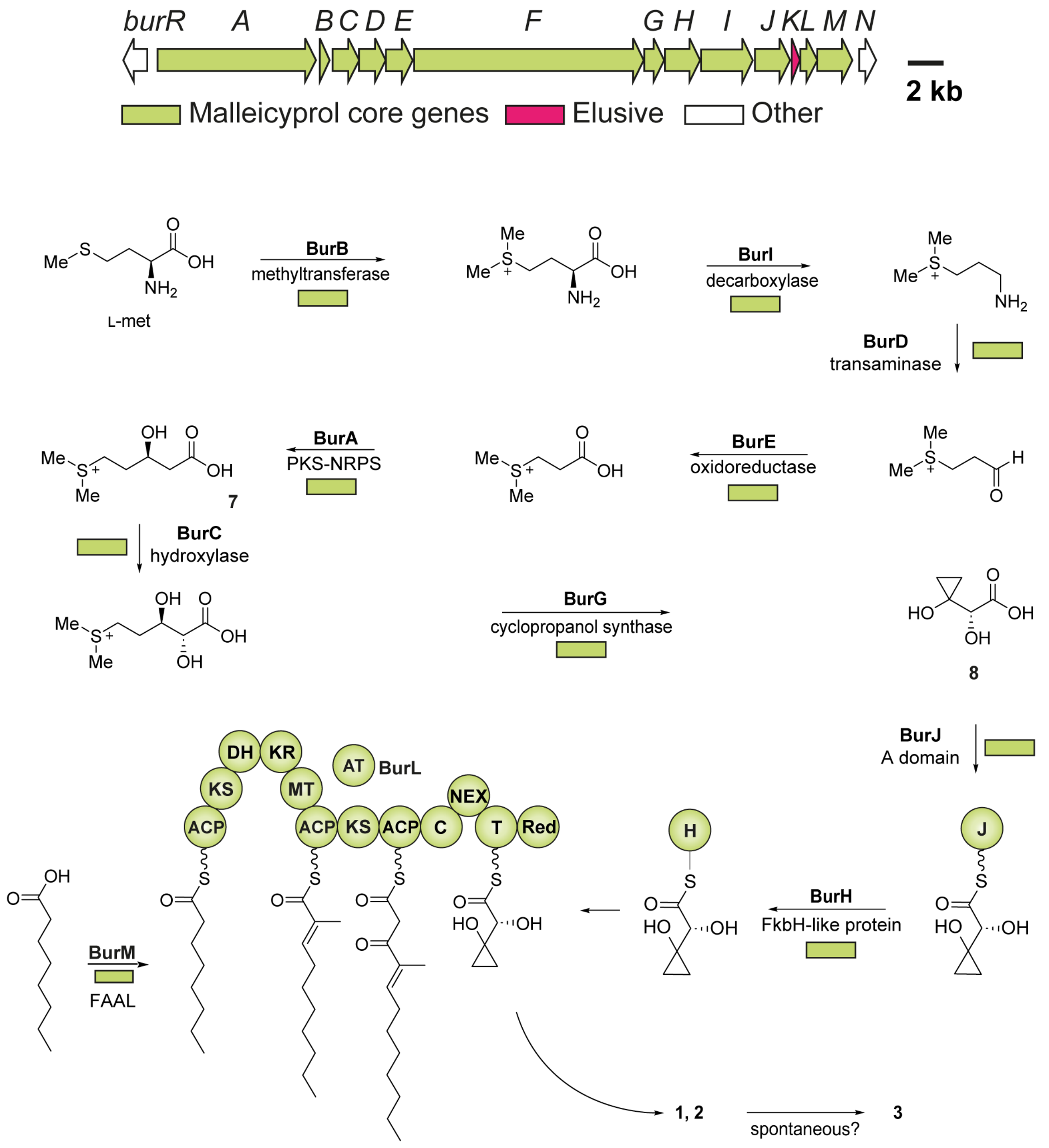

Figure S1. Biosynthesis of malleicyprol. L-methionine is stepwise converted to DMSP by four enzymes (BurBIDE). DMSP is then elongated to *S*-gonyol by the PKS-NRPS hybrid BurA. Hydroxylation (by BurC) and cyclization (by BurG) then generates *R*-trigonic acid. Subsequently, *R*-trigonic acid is loaded onto the BurF-T domain mediated by activation (BurJ) and transfer (BurH) enzymes. Activated octanoic acid (as adenylate, catalyzed by BurM) serves as an additional building block for constructing the final molecule by chain fusion and reductive cyclization. BurL is a predicted *trans* AT that is expected to load the extender unit malonyl-CoA onto the ACP_2 and ACP_3. The origin of **3** has been attributed to be a spontaneous degradation product. PKS-NRPS: polyketide synthase-nonribosomal peptide synthetase; M: module; ACP: acyl carrier protein; KS: ketosynthase; DH: dehydratase; KR: ketoreductase; MT: methyltransferase; AT: acyltransferase; C: condensation domain; T: thiolation domain; Red: reductase; FAAL: fatty acyl adenylate ligase; A domain: adenylation domain; NEX: N-terminal extension. The wavy line of ACP/T domains indicates the phosphopantetheine modification for substrate binding. The straight line of BurH indicates the conserved cysteine residue of the protein containing the thiol group.

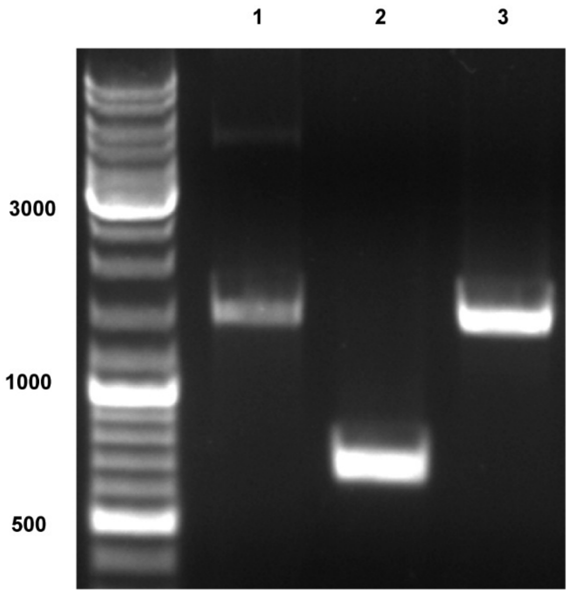

Figure S2. Genetic inactivation of *burK*.M: gene ruler mix. Lane 1: plasmid DNA pJET-*burK*-KO (1,482 bp). Lane 2: genomic DNA of *B. thailandensis* E264 *Pbur* (696 bp). Lane 3: *B. thailandensis* E264 *Pbur*Δ*burK* (1,482 bp).

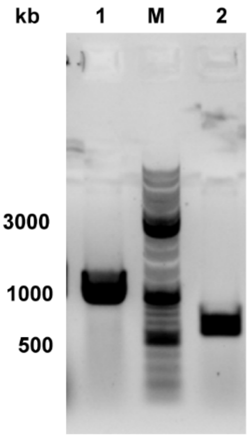

Figure S3. Genetic complementation of *Pbur*Δ*burK*.M: gene ruler mix. Lane 1: plasmid DNA isolated from *Pbur*Δ*burK* pSCrhaB2-*burK* (1,036 bp). Lane 2: plasmid DNA isolated from *Pbur*Δ*burK* pSCrhaB2 (620 bp).

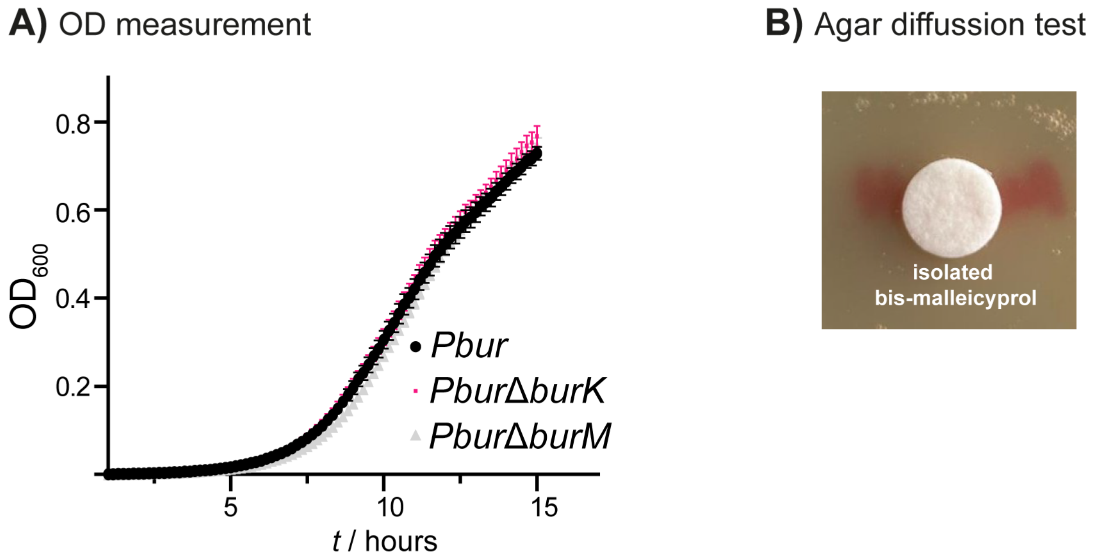

Figure S4. Growth comparison of *Pbur* and *ΔburK* and agar diffusion test.A) The growth of *Pbur* and Δ*burK* were compared by measuring the optical density at 600 nm. The non-producer Δ*burM* served as an additional control. All strains showed an identical growth behavior. B) A paper disc was treated with isolated bis-malleicyprol (30 µg) and placed onto an agar plate previously inoculated with the *Pbur*Δ*burK* strain. The strain grew normally around the disc, indicating no growth inhibition.

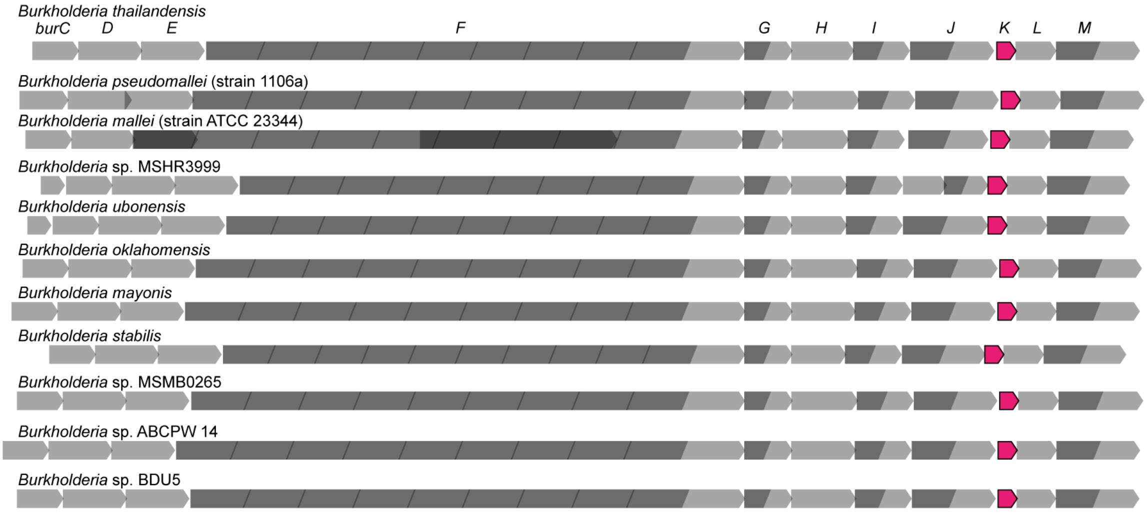

Figure S5. Distribution of *burK* in putative malleicyprol producers.The EFI-GNT webserver was used to analyze the distribution of *burK* genes in *bur* clusters of potential malleicyprol producers. The amino sequence of BurG was used as a query. All gene clusters found harbor a copy of *burK.*

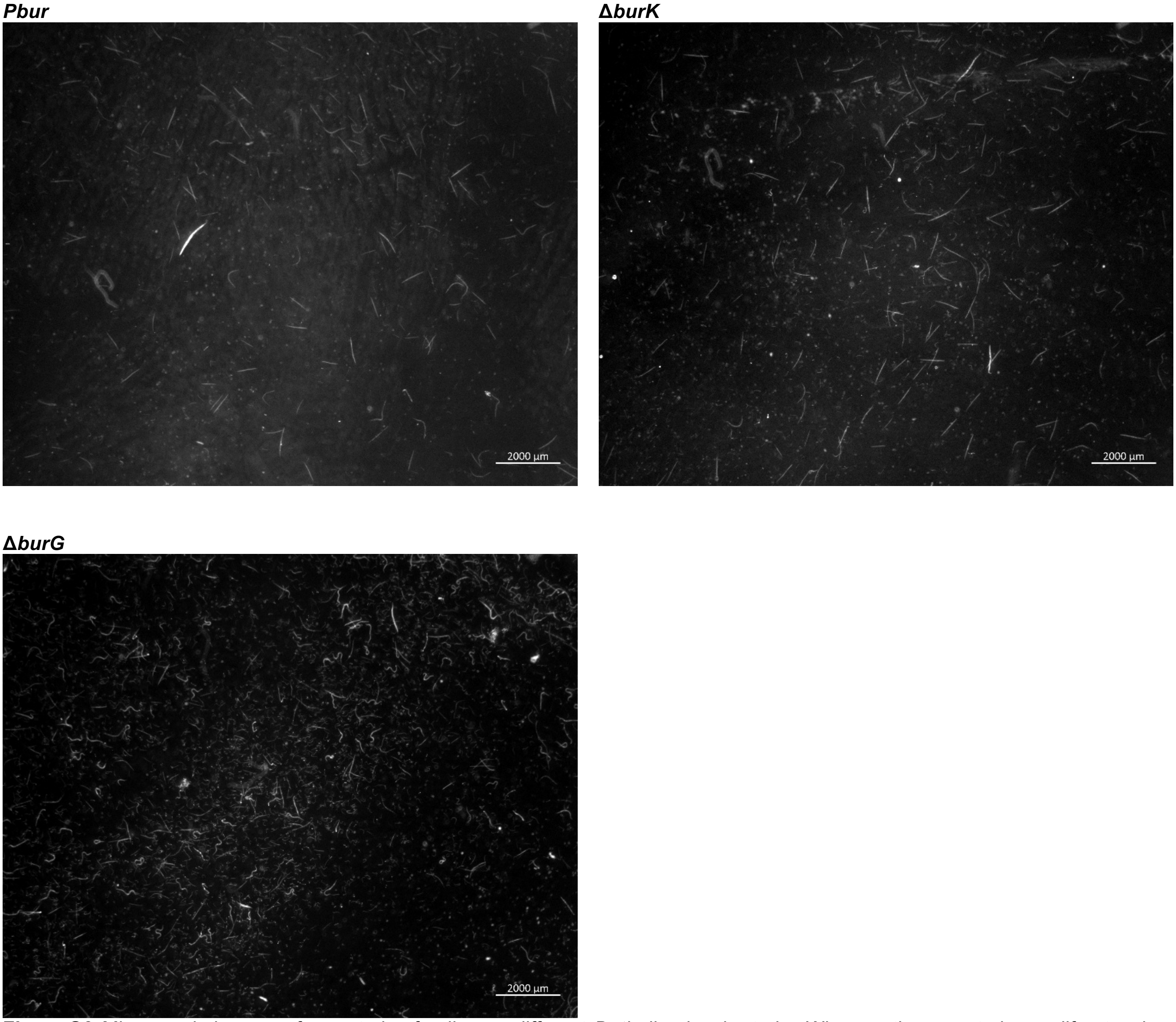

Figure S6. Microscopic images of nematodes feeding on different *B. thailandensis* strains.Whereas the nematodes proliferate when feeding on Δ*burG*, their number did not increase while feeding on *Pbur*. Furthermore, they did not survive the co-incubation. Most nematodes are also dead, when co-incubated with Δ*burK,* but their numbers are increased compared to nematodes when co-incubated with *Pbur*.

A) Composition of BurK

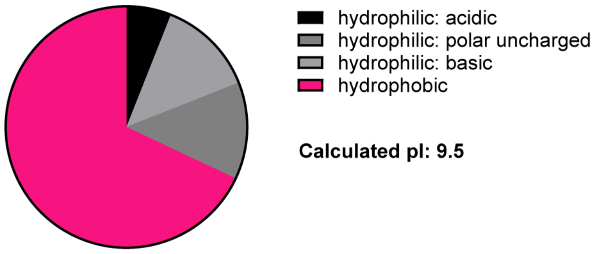

B) TMHMM prediction

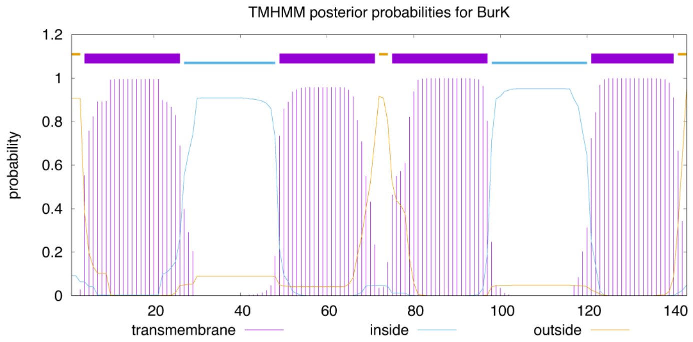

C) Phyre² prediction

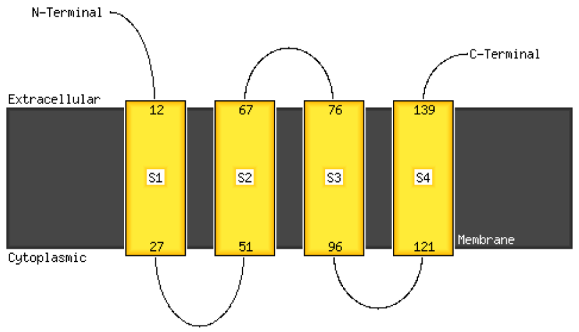

Figure S7. Sequence analyses of BurK.A) BurK is mainly composed of hydrophobic amino acids and has a basic isoelectric point. Both characteristics are commonly found in membrane proteins. B, C) The TMHMM and the Phyre^2 servers both predict that BurK is localized in the cytosolic membrane and contains four transmembrane helices.

A) Model of BurK and comparison to CypB

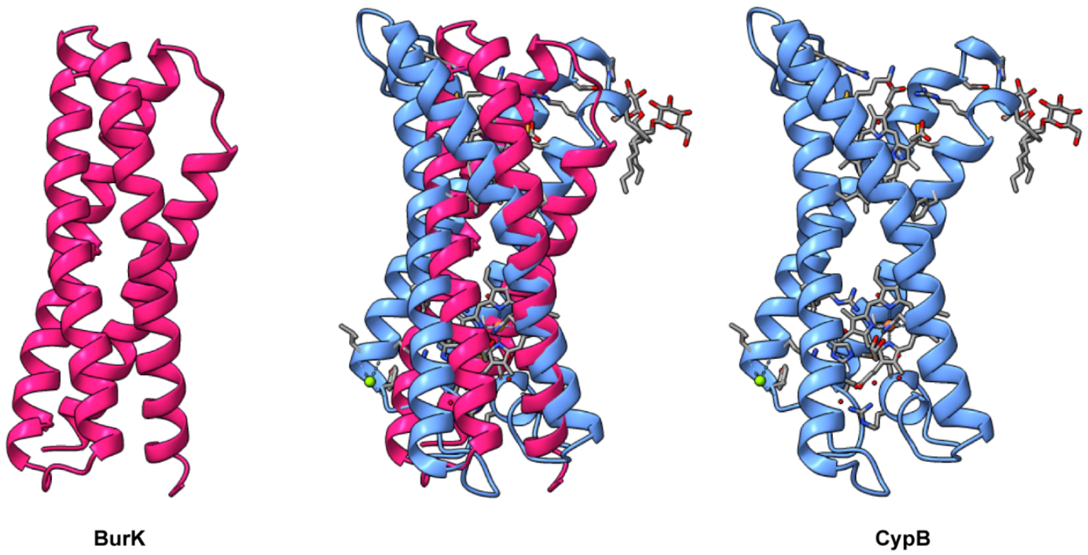

B) Potential heme coordianation sites

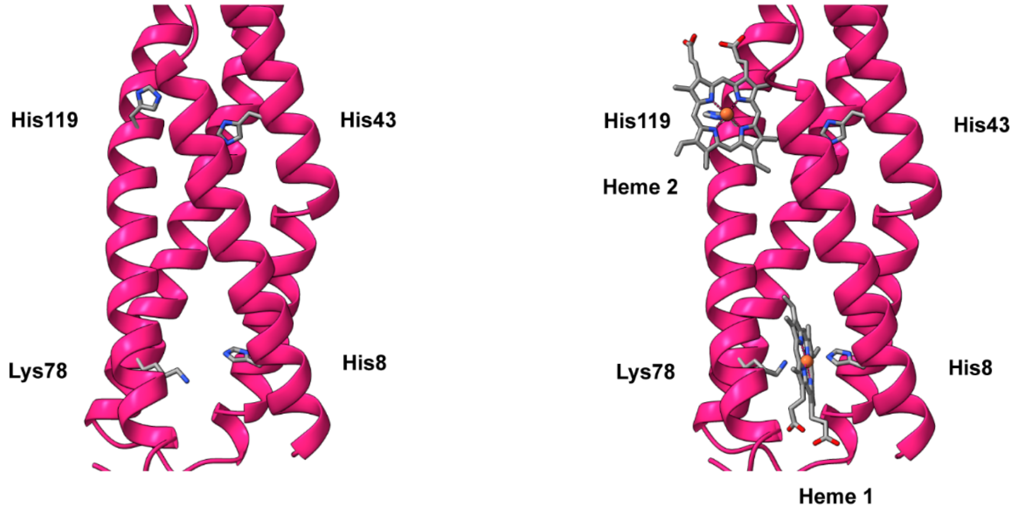

Figure S8. Comparison of BurK and CypB.A) The tertiary structure of BurK was built by AlphaFold3 and compared to the crystallized superoxide oxidase CybB (PDB: 5OC0). Root mean square deviation between 18 pruned atom pairs is 1.303 Å; across all 132 pairs: 7.387 Å). B) The residues that are homologous to CybB are shown. Furthermore, two heme *b* cofactors were integrated into the model using AF3.

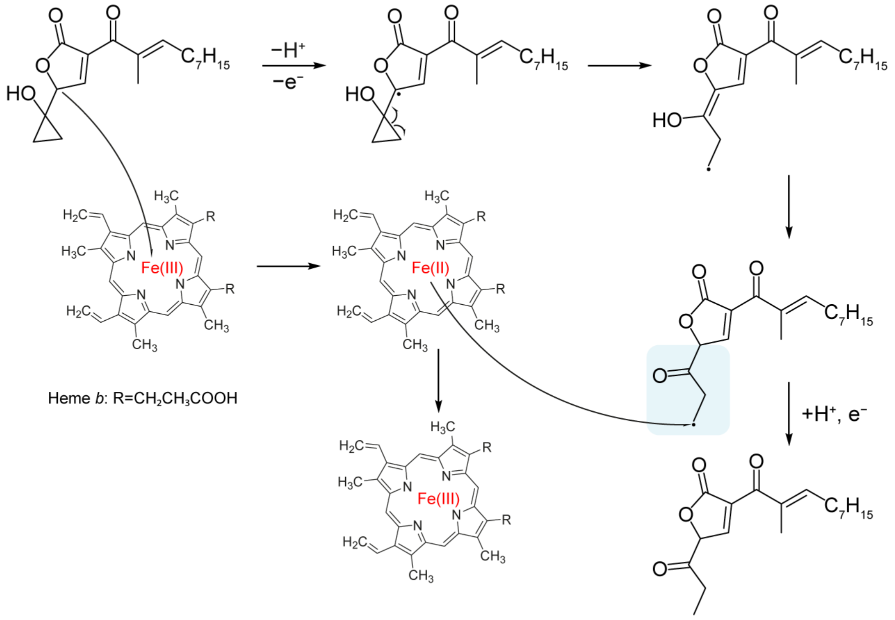

Figure S9. Alternative reaction pathway.BurK could also oxidize the γ-carbon atom of the butenolide ring. Subsequent isomerization would generate the same β-keto radical, as described for the oxidation of the cyclopropanol oxygen. Such reactions are known from synthetic procedures with cyclopropane-containing compounds.

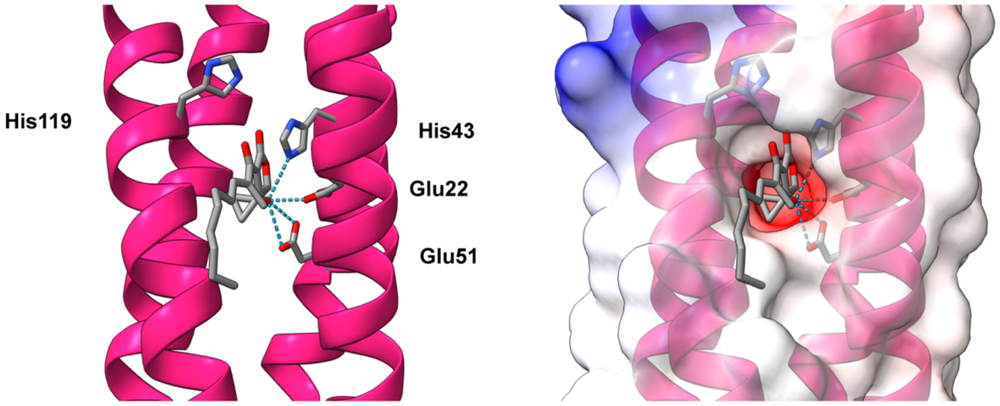

Figure S10. Protein–ligand co-folding model. Malleicyprol (**1**) and BurK were subjected to protein–ligand co-folding modeling using Boltz-2 to predict biomolecular interactions (affinity probability: 0.657, predicted IC50: 1.31 µm). The model suggests that malleicyprol could fit into a negatively charged pocket (indicated by the red shading) next to residues that are assumed to coordinate the heme 2 cofactor. There, a glutamic acid residue might also facilitate proton shuttling.

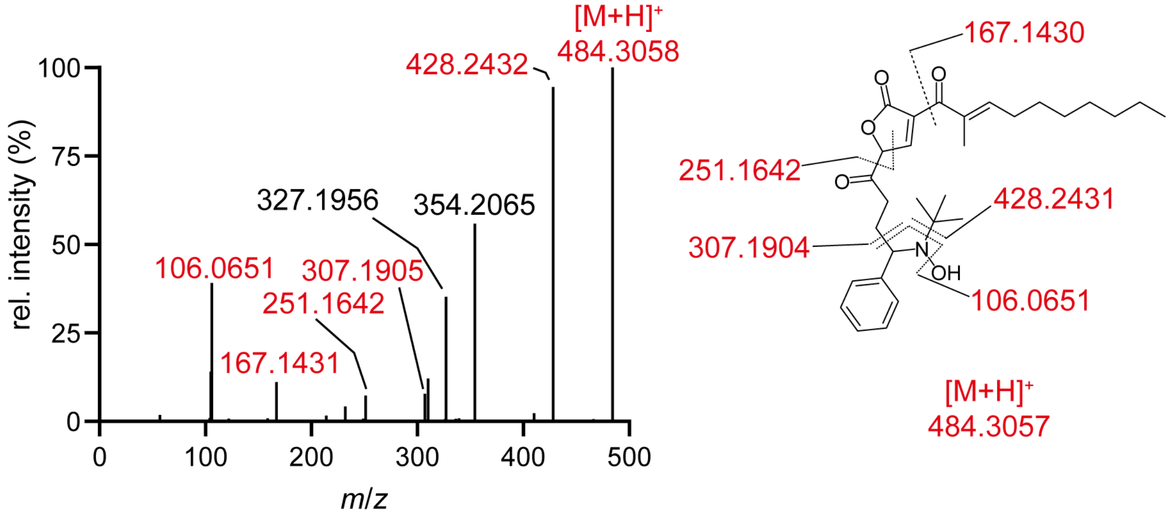

Figure S11. MS^2 analysis of PBN-adduct.The compound **7** (*m*/*z* 484.3057 [M + H]$^+$) was analyzed using MS^2 (normalized collision energy = 15 %). The calculated *m*/*z* values are indicated at the structure. Red values indicate identified fragments.

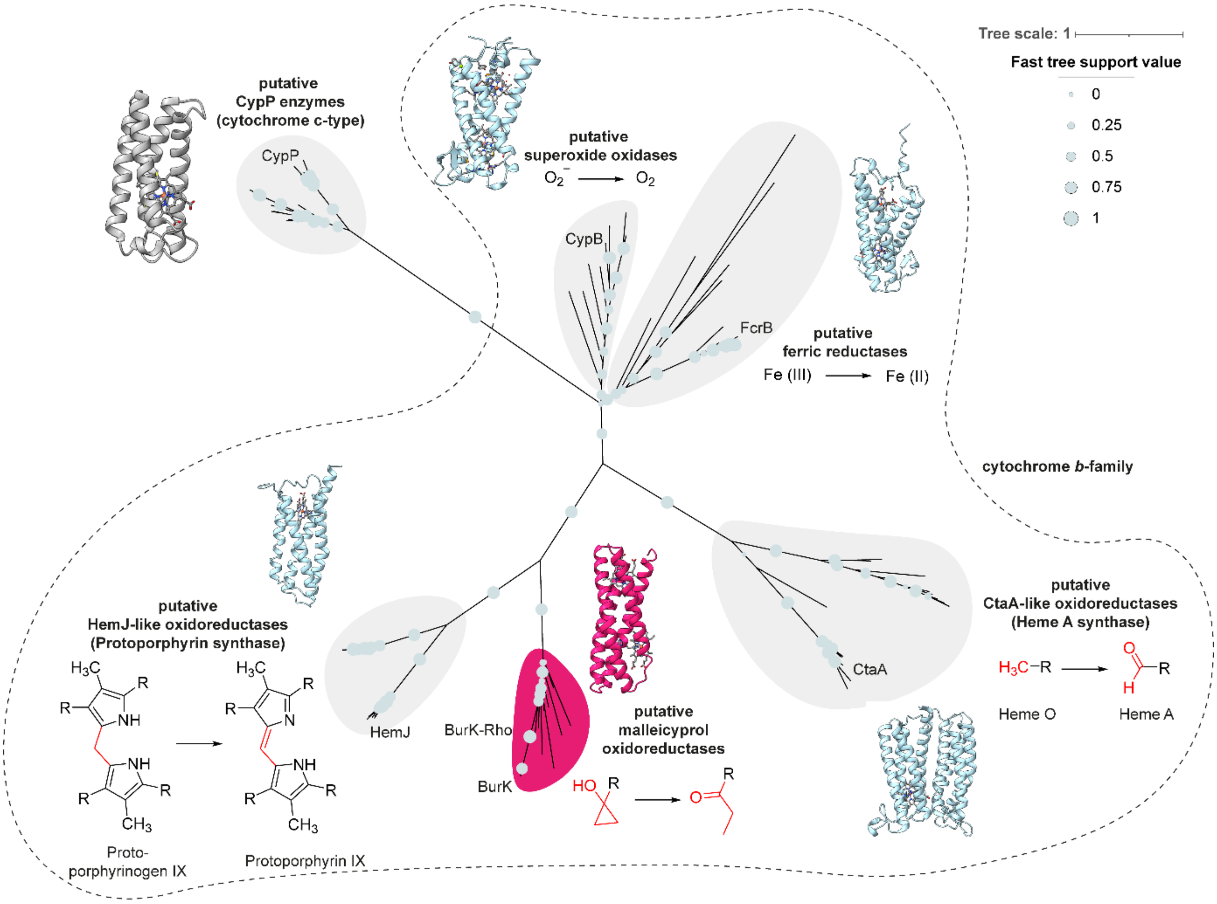

Figure S12. Phylogeny of BurK and related cytochrome *b* enzymes.BurK was aligned with structurally related oxidoreductases of the cytochrome *b* family. All are heme-dependent enzymes that catalyze redox reactions. CypP-like enzymes (cytochrome *c*-type, also containing of four α-helices and heme-dependent) were selected as outgroup. Protein structures were either obtained from the PDB (CypP: 6A3K, CtaA: 6A2J, CybB: 5OC0) or generated by AF3 (FcrB: WP_011086339, HemJ: WP_023003745). The respective enzymatic reactions are shown.

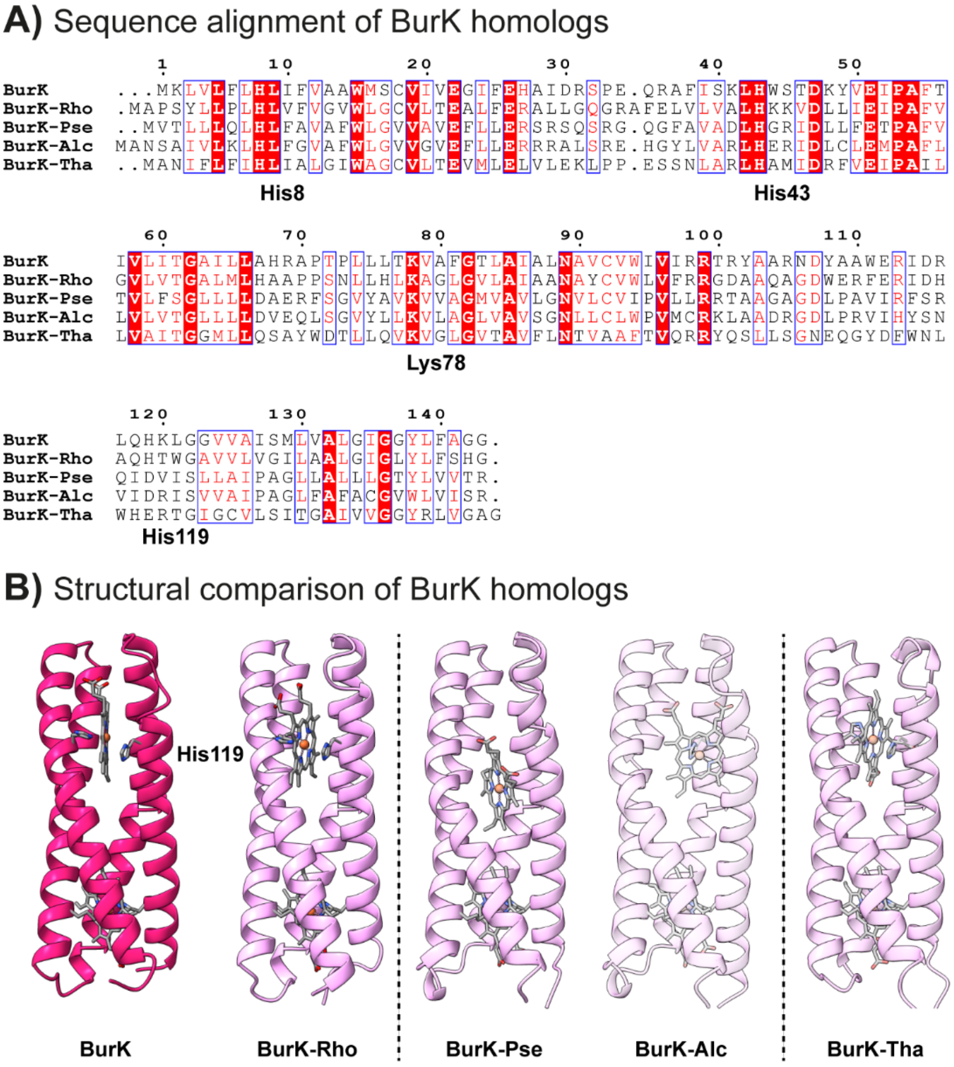

Figure S13. Sequence comparison of BurK-like enzymes. A) The tested BurK-like proteins were aligned with BurK. The conserved residues that were mutated in BurK are indicated. The residues H8, H43 and K78 are conserved between the proteins. The H119 residue is only present in the strongly active BurK and BurK-Rho variants in contrast to BurK-Pse and BurK-Alc. B) All proteins were modeled using AF3. Comparison of the models show that the H119 residue is differently orientated in BurK-Tha.

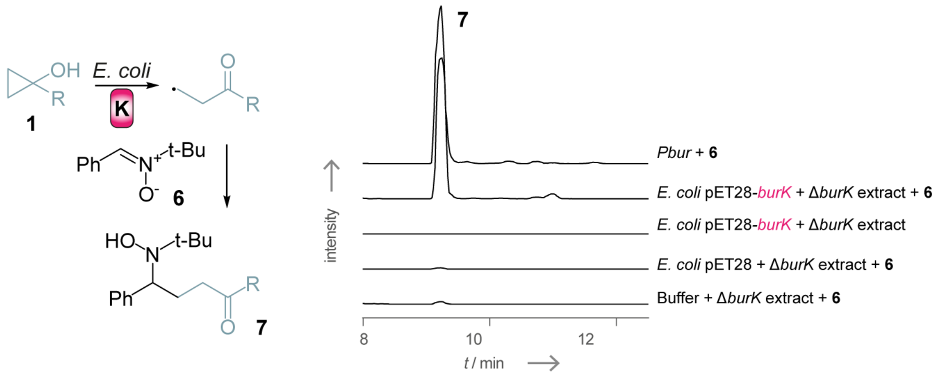

Figure S14. Radical-trapping experiment using *E. coli* strains.Incubation of the *E. coli* strain carrying burK, a Δ*burK* extract, and PBN results in the formation of compound **7**, consistent with the experiments carried out in *B. thailandensis*.

Supplementary tables

Table S1. Transcriptome analysis.The expression of *bur* genes is induced during infection. Data obtained from published comparative transcriptome analysis. RPM: reads per million.

Gene	Enzyme function	Infected mice (RPM)	Soil medium (RPM)	log_2 Fold Change	*p*-value
burH	Trigonic acid transfer	749	21	5.19	1.19E-14
burG	Trigonic acid synthesis	879	26	5.11	1.12E-10
burF	Malleicyprol synthesis	748	23	5.00	4.37E-11
burL	Putative *trans*-acyl transferase	574	21	4.78	1.82E-12
burK	**Unknown**	**534**	**20**	**4.76**	**1.75E-14**

Table S2. Transposon analysis.Transposon inactivation of *burK* leads to reduced abundance of the strains after lung colonization compared to the initial population. Only genes that meet a 3-fold cutoff criterion are shown. The *burK* transposon insertion ranks among the top 2 % of genes with the highest influence (rank 35, out of a total of 1,455 genes). Data obtained from published transposon-sequencing study.

Gene	Enzyme function	Fold Change	Input-Means	Lung-Output-Means	Rank	ID
burK	**Unknown**	**−177.48**	**159.68**	**0.90**	**35**	**BP1026B_II0330**
burN	Putative transporter	−9.64	41.63	4.32	708	BP1026B_II0327
burB	DMSP synthesis	−6.75	134.06	19.84	872	BP1026B_II0339
burE	DMSP synthesis	−4.71	49.24	10.45	1100	BP1026B_II0336
burM	Octanoic acid activation	−3.57	78.97	22.15	1308	BP1026B_II0331

Table S3. Analyzed BurK-like proteins.The Uniprot accession numbers are indicated. Cross-complementation of *Pbur*Δ*burK* with the corresponding gene of the BurK ortholog of *Rhodoferax sediminis* leads to the highest product formation of burkholderic acid.

Organism	Abbreviation	Accession number
Pseudomonas denitrificans	BurK-Pse	A0A9X7R4C8
Alcanivorax sp. MD8A	BurK-Alc	A0A2N8M182
Rhodoferax sediminis	**BurK-Rho**	**A0A515EK06**
Thalassolituus maritimus	BurK-Tha	A0A1N7LSZ2

Table S4. Protein accession numbers of proteins used for the phylogeny.

Accession number	Organism	Putative function
**BurK** Q2T3G4	*Burkholderia thailandensis*	Malleicyprol oxidoreductase
A2S1X8	*Burkholderia mallei*	Malleicyprol oxidoreductase
A0A069BD99	*Burkholderia pseudomallei*	Malleicyprol oxidoreductase
A0A1B4FVJ4	*Burkholderia mayonis*	Malleicyprol oxidoreductase
**BurK-Rho** A0A515EK06	*Rhodoferax sediminis*	Malleicyprol oxidoreductase
A0A315CJV3	*Limnohabitans* sp.	Malleicyprol oxidoreductase
A0A5C9CUR5	*Comamonadaceae* bacterium	Malleicyprol oxidoreductase
A0A1D9H493	*Cupriavidus* sp.	Malleicyprol oxidoreductase
A0A963Q1A7	*Ottowia* sp.	Malleicyprol oxidoreductase
A0A962KGG7	*Halioglobus* sp.	Malleicyprol oxidoreductase
A0A975CER3	*Ottowia testudinis*	Malleicyprol oxidoreductase
A0A1V3RPJ8	*Hydrogenophaga* sp.	Malleicyprol oxidoreductase
A0A495HXS9	*Acidovorax* sp.	Malleicyprol oxidoreductase
**HemJ** WP_193600755.1	*Cereibacter johrii*	Protoporphyrin synthase
WP_097030061.1	*Cereibacter ovatus*	Protoporphyrin synthase
WP_151718938.1	*Gemmobacter serpentinus*	Protoporphyrin synthase
WP_335424176.1	*Gemmobacter denitrificans*	Protoporphyrin synthase
MDZ7909284.1	*Gemmobacter* sp.	Protoporphyrin synthase
WP_298566899.1	*Aliiroseovarius* sp.	Protoporphyrin synthase
WP_242114107.1	*Aliiroseovarius sediminis*	Protoporphyrin synthase
WP_304880076.1	*Pseudotabrizicola* sp.	Protoporphyrin synthase
MFN3722447.1	*Paracoccaceae* sp.	Protoporphyrin synthase
MCU0828611.1	*Tabrizicola* sp.	Protoporphyrin synthase
WP_301793030.1	*Burkholderia oklahomensis*	Protoporphyrin synthase
EIP88156.1	*Burkholderia humptydooensis*	Protoporphyrin synthase
WP_179404833.1	*Burkholderia guangdongensis*	Protoporphyrin synthase
WP_328700027.1	*Burkholderia glumae*	Protoporphyrin synthase
WP_407853051.1	*Burkholderia gladioli*	Protoporphyrin synthase
WP_411187086.1	*Paraburkholderia* sp.	Protoporphyrin synthase
WP_121277198.1	*Trinickia fusca*	Protoporphyrin synthase
WP_102129763.1	*Burkholderia* sp.	Protoporphyrin synthase
WP_120289555.1	*Paraburkholderia* sp.	Protoporphyrin synthase
CAH2927318.1	*Paraburkholderia* sp.	Protoporphyrin synthase
**CypB** NP_415935.2	*Escherichia coli*	Superoxide oxidase
YP_403372.2	*Shigella dysenteriae*	Superoxide oxidase
NP_460598.1	*Salmonella enterica*	Superoxide oxidase
YP_003612579.1	*Enterobacter cloacae*	Superoxide oxidase
YP_002348217.1	*Yersinia pestis*	Superoxide oxidase
NP_416483.4	*Escherichia coli*	Superoxide oxidase
WP_002211611.1	*Yersinia pestis*	Superoxide oxidase
NP_460220.1	*Salmonella enterica*	Superoxide oxidase
NP_249609.1	*Pseudomonas aeruginosa*	Superoxide oxidase
NP_105468.1	*Mesorhizobium loti*	Superoxide oxidase
NP_518320.1	*Ralstonia solanacearum*	Superoxide oxidase

**FcrB** NP_770197.1	*Bradyrhizobium diazoefficiens*	Ferric reductase
NP_207427.1	*Helicobacter pylori* 26695	Ferric reductase
ZP_01313790.1	*Desulfuromonas acetoxidans*	Ferric reductase
NP_440882.1	*Synechocystis* sp. PCC 6803	Ferric reductase
NP_718925.1	*Shewanella oneidensis*	Ferric reductase
AP_002041.1	*Escherichia coli*	Ferric reductase
WP_304552353.1	*Bradyrhizobium* sp.	Ferric reductase
WP_271529189.1	*Bradyrhizobium* sp.	Ferric reductase
WP_319335045.1	*Bradyrhizobium* sp.	Ferric reductase
WP_253710724.1	*Bradyrhizobium* sp.	Ferric reductase
HEY8333971.1	*Tardiphaga* sp.	Ferric reductase
WP_265081761.1	*Rhodopseudomonas* sp.	Ferric reductase
WP_011473175.1	*Rhodopseudomonas palustris*	Ferric reductase
WP_044410318.1	*Rhodopseudomonas* sp.	Ferric reductase
WP_322514587.1	*Rhodopseudomonas palustris*	Ferric reductase
WP_238738162.1	*Rhodopseudomonas infernalis*	Ferric reductase
YP_002907809.1	*Burkholderia glumae*	Ferric reductase
YP_222970.1	*Brucella abortus*	Ferric reductase
YP_002002877.1	*Neisseria gonorrhoeae*	Ferric reductase
**CtaA** WP_003329100.1	*Bacillus atrophaeus*	Heme A synthase
WP_268294403.1	*Bacillus inaquosorum*	Heme A synthase
WP_268419165.1	*Bacillus inaquosorum*	Heme A synthase
WP_269775142.1	*Bacillus siamensis*	Heme A synthase
WP_006638310.1	*Bacillus* sp.	Heme A synthase
WP_175076863.1	*Bacillus* sp.	Heme A synthase
TWL27218.1	*Bacillus licheniformis*	Heme A synthase
WP_026586837.1	*Bacillus* sp.	Heme A synthase
WP_353855885.1	*Bacillus maqinnsis*	Heme A synthase
SPU05312.1	*Bacillus tequilensis*	Heme A synthase
HEY2979336.1	*Burkholderiaceae*	Heme A synthase
HWL72545.1	*Burkholderia* sp.	Heme A synthase
MBA3253781.1	*Burkholderiaceae*	Heme A synthase
HKO66542.1	*Burkholderiaceae*	Heme A synthase
MGH6611184.1	*Burkholderiaceae*	Heme A synthase
MBA2411343.1	*Burkholderiaceae*	Heme A synthase
MGB2815837.1	*Burkholderiaceae*	Heme A synthase
MDO5057954.1	*Lautropia sp.*	Heme A synthase
WP_323472475.1	*Polynucleobacter* sp.	Heme A synthase
WP_215352030.1	*Polynucleobacter* sp.	Heme A synthase
**CypP** 6A3L_A	*Shewanella violacea*	CypP-like cytochrome *c*
WP_287947399.1	*Shewanella* sp.	CypP-like cytochrome *c*
MEI6860269.1	*Shewanella* sp.	CypP-like cytochrome *c*
WP_142872414.1	*Shewanella* sp.	CypP-like cytochrome *c*
WP_269971691.1	*Shewanella* sp.	CypP-like cytochrome *c*
WP_144042772.1	*Shewanella hanedai*	CypP-like cytochrome *c*
WP_394391993.1	*Shewanella woodyi*	CypP-like cytochrome *c*
WP_173293848.1	*Shewanella* sp. VB17	CypP-like cytochrome *c*

WP_126519936.1	*Shewanella canadensis*	CypP-like cytochrome *c*
WP_299794165.1	*Shewanella atlantica*	CypP-like cytochrome *c*
MBK9137035.1	Betaproteobacterium	CypP-like cytochrome *c*
HEY4068925.1	Burkholderiaceae bacterium	CypP-like cytochrome *c*
HET7865801.1	Burkholderiaceae bacterium	CypP-like cytochrome *c*
MGL4575421.1	Burkholderiaceae bacterium	CypP-like cytochrome *c*
MBW8759815.1	Burkholderiaceae bacterium	CypP-like cytochrome *c*
HEY9029101.1	Burkholderiaceae bacterium	CypP-like cytochrome *c*
WP_215363897.1	*Polynucleobacter* sp. es-EL-1	CypP-like cytochrome *c*
WP_255535964.1	*Polynucleobacter* sp. MG-27-Goln-C1	CypP-like cytochrome *c*
WP_293627675.1	*Polynucleobacter* sp.	CypP-like cytochrome *c*
WP_088527051.1	*Polynucleobacter* sp.	CypP-like cytochrome *c*

Table S5. Strains used in this study.

Strains	Relevant genotype and descriptions	Source
Escherichia coli TOP10	General cloning strain	Invitrogen
Escherichia coli Lemo BL21(DE3)	Protein production strain	NEB
Escherichia coli OP50	Nematode food source	[21]
Caenorhabditis elegans	Strain N2 (var. Bristol)	[21]
Burkholderia thailandensis E264 *Pbur*	Promotor of *burA* was exchanged with Δ*PthaA*; Tet^R	[5]
B. thailandensis E264 *Pbur*Δ*burG*	Kan^R cassette inserted into *burG*; Tet^R, Kan^R	[23]
B. thailandensis E264 *Pbur*Δ*burM*	Kan^R cassette inserted into *burM*; Tet^R, Kan^R	[24]
B. thailandensis E264 *Pbur*Δ*burK*	Kan^R cassette inserted into *burK*; Tet^R, Kan^R	This study
B. thailandensis E264 *Pbur*Δ*burK* pSCrhaB2	*B. thailandensis* E264 *Pbur*Δ*burK* carrying pSCrhaB2	This study
B. thailandensis E264 *Pbur*Δ*burK* pSCrhaB2-*burK*	*B. thailandensis* E264 *Pbur*Δ*burK* carrying pSCrhaB2-*burK*	This study
B. thailandensis E264 *Pbur*Δ*burK* pSCrhaB2-*burK*-H8A	*B. thailandensis* E264 *Pbur*Δ*burK* carrying pSCrhaB2-*burK*-H8A	This study
B. thailandensis E264 *Pbur*Δ*burK* pSCrhaB2-*burK*-H43A	*B. thailandensis* E264 *Pbur*Δ*burK* carrying pSCrhaB2-*burK*-H43A	This study
B. thailandensis E264 *Pbur*Δ*burK* pSCrhaB2-*burK*-K78A	*B. thailandensis* E264 *Pbur*Δ*burK* carrying pSCrhaB2-*burK*-K78A	This study
B. thailandensis E264 *Pbur*Δ*burK* pSCrhaB2-*burK*-H119A	*B. thailandensis* E264 *Pbur*Δ*burK* carrying pSCrhaB2-*burK*-H119A	This study
B. thailandensis E264 *Pbur*Δ*burK* pSCrhaB2-*burK*-Pse	*B. thailandensis* E264 *Pbur*Δ*burK* carrying pSCrhaB2-*burK*-Pden	This study
B. thailandensis E264 *Pbur*Δ*burK* pSCrhaB2-*burK*-Alc	*B. thailandensis* E264 *Pbur*Δ*burK* carrying pSCrhaB2-*burK*-Alca	This study
B. thailandensis E264 *Pbur*Δ*burK* pSCrhaB2-*burK*-Rho	*B. thailandensis* E264 *Pbur*Δ*burK* carrying pSCrhaB2-*burK*-Rho	This study
B. thailandensis E264 *Pbur*Δ*burK* pSCrhaB2-*burK*-Tha	*B. thailandensis* E264 *Pbur*Δ*burK* carrying pSCrhaB2-*burK*-Tha	This study
Escherichia coli TOP10 pET28-*burK*	Cloning of pET28-*burK*	This study
Escherichia coli Lemo BL21(DE3) pET28-*burK*	Production of BurK with *C*-terminal His_6-Tag	This study
Escherichia coli Lemo BL21(DE3) pET28	*Escherichia coli* Lemo BL21(DE3) carrying pET28 (empty-vector control)	This study

Table S6. Plasmids used in this study.

Plasmid	Description	Source
pJET1.2	Subcloning vector for blunt-end amplified DNA after PCR	Thermo Fisher Scientific
pET28a	Expression vector for *N*- or *C*-terminally His_6-tagged proteins	Novagen
pSCrhaB2	Low-copy vector with rhamnose-inducible promotor; $Trim^R$	[2]
pJET-*burK*-KO	pJET1.2 containing parts of *burJ* and *burL*; Kan^R	This study
pSCrhaB2-*burK*	Complementation vector for *burK*	This study
pSCrhaB2-*burK*-H8A	Complementation vector encoding BurK H8A mutant	This study
pSCrhaB2-*burK*-H43A	Complementation vector encoding BurK H43A mutant	This study
pSCrhaB2-*burK*-K78A	Complementation vector encoding BurK K78A mutant	This study
pSCrhaB2-*burK*-H119A	Complementation vector encoding BurK H119A mutant	This study
pSCrhaB2-*burK*-Pse	Complementation vector encoding BurK homolog from *Pseudomonas denitrificans*	This study
pSCrhaB2-*burK*-Alc	Complementation vector encoding BurK homolog from *Alcanivorax* sp. MD8A	This study
pSCrhaB2-*burK*-Rho	Complementation vector encoding BurK homolog from *Rhodoferax sediminis*	This study
pSCrhaB2-*burK*-Tha	Complementation vector encoding BurK homolog from *Thalassolituus maritimus*	This study
pET28-*burK*	Expression vector of *burK* with *C*-terminal His_6-Tag	This study
pET28-*burK*-Pse	Cloning of pSCrhaB2-*burK*-Pse	Synthetic origin
pET28-*burK*-Alc	Cloning of pSCrhaB2-*burK*-Alc	Synthetic origin
pET28-*burK*-Rho	Cloning of pSCrhaB2-*burK*-Rho	Synthetic origin
pET28-*burK*-Tha	Cloning of pSCrhaB2-*burK*-Tha	Synthetic origin

Table S7. Oligonucleotide primers used in this study.Restriction endonuclease recognition sites and homology regions are underlined. Codons that were used to introduce site-directed mutations are indicated in bold (GCC). Artificial base pairs that were introduced to maintain the correct reading frame are highlighted in italics.

Name	Sequence (5′-3′)	Use
T7 Seq F	TAATACGACTCACTATAGGG	pET28a sequencing
pJET1.2 forward	CGACTCACTATAGGGAGAGCGGC	pJET1.2 sequencing
pJET1.2 reverse	AAGAACATCGATTTTCCATGGCAG	pJET1.2 sequencing
pSCrhabB2_fw	GCCTCCTGATGTCGTCAACA	pSCrhabB2 sequencing
pSCrhabB2_rv	TGTCCTACTCAGGAGAGCGTTCAC	pSCrhabB2 sequencing
*burK*_KO1_F	GATGGCTCGAGTTTTTCAGCAAGATCCCCGATCCCGATCTCGC	Cloning of pJET-*burK*-KO
*burK*_KO1_R	CGTCAGACCCCGTTGGCGTCTCCTTCATGTTCG	Cloning of pJET-*burK*-KO
*burK*_KO2_F	GCACATTTCCCCGCGACGCCGCTGTTGCT	Cloning of pJET-*burK*-KO
*burK*_KO2_R	ATTGTAGGAGATCTTCTAGAAAGATGACGCGTGAGAATCCGGC	Cloning of pJET-*burK*-KO
*kan*_F	GAAGGAGACGCCAACGGGGTCTGACGCTCA	Cloning of pJET-*burK*-KO
*kan*_R	AACAGCGGCGTCGCGGGGAAATGTGCGCG	Cloning of pJET-*burK*-KO
*burK*_KO_screening_fw	GGGCGGCGAGAACATCTACC	Screening of *B. thailandensis* E264 *Pbur*Δ*burK*
*burK*_KO_screening_fw	CGGATCACGATCCACACGCA	Screening of *B. thailandensis* E264 *Pbur*Δ*burK*
*burK*_compl_F	GGTCATATGAAGCTTGTGCTGTTCCTG	Cloning of pSCrhaB2-*burK*
*burK*_compl_R	GGTGGATCCTCATCCTCCTGCGAACAGG	Cloning of pSCrhaB2-*burK*
*burK*_H8A_fw	GTGCTGTTCCTG**GCC**CTGATTTTCGTC	SDM of pSCrhaB2-*burK*
*burK*_H8A_rv	AAGCTTCATATGTGATCCTGC	SDM of pSCrhaB2-*burK*
*burK*_H43A_fw	ATCTCGAAGCTG**GCC**TGGAGCACCGAC	SDM of pSCrhaB2-*burK*
*burK*_H43A_rv	GAACGCGCGTTGCTCGGG	SDM of pSCrhaB2-*burK*
*burK*_K78A_fw	CTGTTGCTGACC**GCC**GTCGCGTTCGGC	SDM of pSCrhaB2-*burK*
*burK*_K78A_rv	CGGCGTCGGCGCGCGGTG	SDM of pSCrhaB2-*burK*
*burK*_H119A_fw	GACCGCCTGCAG**GCC**AAGCTCGGCGGT	SDM of pSCrhaB2-*burK*
*burK*_H119A_rv	GATGCGCTCCCACGCCGC	SDM of pSCrhaB2-*burK*
*burK*_Pse_fw	AATTCAGCAGGATCACATATGGTGACCTTGCTCCTG	Cloning of pSCrhaB2-*burK*-Pse
*burK*_Pse_rv	TGCAGGTCGACTCTAGAGTCAGCGAGTGACCACCAG	Cloning of pSCrhaB2-*burK*-Pse
*burK*_Alc_fw	AATTCAGCAGGATCACATATGGCGAATAGCGCAATC	Cloning of pSCrhaB2-*burK*-Alc
*burK*_Alc_rv	TGCAGGTCGACTCTAGAGCTAGCGACTGATCACCAG	Cloning of pSCrhaB2-*burK*-Alc
*burK*_Rho_fw	AATTCAGCAGGATCACATATGGCGCCTTCTTACCTG	Cloning of pSCrhaB2-*burK*-Rho
*burK*_Rho_rv	TGCAGGTCGACTCTAGAGTTAGCCGTGGCTAAACAG	Cloning of pSCrhaB2-*burK*-Rho
*burK*_Tha_fw	AATTCAGCAGGATCACATATGGCGAACATTTTTCTA	Cloning of pSCrhaB2-*burK*-Tha
*burK*_Tha_rv	TGCAGGTCGACTCTAGAGTCAACCTGCCCCCACGAG	Cloning of pSCrhaB2-*burK*-Tha
*burK*_recom_F_*Nco*I	GGTCCATGG*CG*AAGCTTGTGCTGTTCCTG	Cloning of pET28-*burK*
*burK*_recom_R_*Xho*I	GGTCTCGAGTCCTCCTGCGAACAGG	Cloning of pET28-*burK*

References

[1] M. Green, J. Sambrook, *Molecular cloning: A laboratory manual*, Cold Spring Harbor Laboratory Press, Cold Spring Harbor, **2012**.
[2] S. T. Cardona, M. A. Valvano, *Plasmid* **2005**, *54*, 219-228.
[3] G. Bertani, *J. Bacteriol.* **1951**, *62*, 293–300.
[4] K. H. Choi, A. Kumar, H. P. Schweizer, *J. Microbiol. Methods* **2006**, *64*, 391-397.
[5] J. Franke, K. Ishida, C. Hertweck, *Angew. Chem. Int. Ed.* **2012**, *51*, 11611-11615.
[6] M. C. Chambers, B. Maclean, R. Burke, D. Amodei, D. L. Ruderman, S. Neumann, L. Gatto, B. Fischer, B. Pratt, J. Egertson, K. Hoff, D. Kessner, N. Tasman, N. Shulman, B. Frewen, T. A. Baker, M.-Y. Brusniak, C. Paulse, D. Creasy, L. Flashner, K. Kani, C. Moulding, S. L. Seymour, L. M. Nuwaysir, B. Lefebvre, F. Kuhlmann, J. Roark, P. Rainer, S. Detlev, T. Hemenway, A. Huhmer, J. Langridge, B. Connolly, T. Chadick, K. Holly, J. Eckels, E. W. Deutsch, R. L. Moritz, J. E. Katz, D. B. Agus, M. MacCoss, D. L. Tabb, P. Mallick, *Nat. Biotechnol.* **2012**, *30*, 918-920.
[7] R. Schmid, S. Heuckeroth, A. Korf, A. Smirnov, O. Myers, T. S. Dyrlund, R. Bushuiev, K. J. Murray, N. Hoffmann, M. Lu, A. Sarvepalli, Z. Zhang, M. Fleischauer, K. Dührkop, M. Wesner, S. J. Hoogstra, E. Rudt, O. Mokshyna, C. Brungs, K. Ponomarov, L. Mutabdžija, T. Damiani, C. J. Pudney, M. Earll, P. O. Helmer, T. R. Fallon, T. Schulze, A. Rivas-Ubach, A. Bilbao, H. Richter, L.-F. Nothias, M. Wang, M. Orešič, J.-K. Weng, S. Böcker, A. Jeibmann, H. Hayen, U. Karst, P. C. Dorrestein, D. Petras, X. Du, T. Pluskal, *Nat. Biotechnol.* **2023**, *41*, 447-449.
[8] E. Gasteiger, A. Gattiker, C. Hoogland, I. Ivanyi, R. D. Appel, A. Bairoch, *Nucleic Acids Res.* **2003**, *31*, 3784-3788.
[9] L. A. Kelley, S. Mezulis, C. M. Yates, M. N. Wass, M. J. E. Sternberg, *Nat. Protoc.* **2015**, *10*, 845-858.
[10] A. Krogh, B. Larsson, G. von Heijne, E. L. Sonnhammer, *J. Mol. Biol.* **2001**, *305*, 567-580.
[11] L. Zimmermann, A. Stephens, S.-Z. Nam, D. Rau, J. Kübler, M. Lozajic, F. Gabler, J. Söding, A. N. Lupas, V. Alva, *J. Mol. Biol.* **2018**, *430*, 2237-2243.
[12] J. Abramson, J. Adler, J. Dunger, R. Evans, T. Green, A. Pritzel, O. Ronneberger, L. Willmore, A. J. Ballard, J. Bambrick, S. W. Bodenstein, D. A. Evans, C.-C. Hung, M. O'Neill, D. Reiman, K. Tunyasuvunakool, Z. Wu, A. Žemgulytė, E. Arvaniti, C. Beattie, O. Bertolli, A. Bridgland, A. Cherepanov, M. Congreve, A. I. Cowen-Rivers, A. Cowie, M. Figurnov, F. B. Fuchs, H. Gladman, R. Jain, Y. A. Khan, C. M. R. Low, K. Perlin, A. Potapenko, P. Savy, S. Singh, A. Stecula, A. Thillaisundaram, C. Tong, S. Yakneen, E. D. Zhong, M. Zielinski, A. Žídek, V. Bapst, P. Kohli, M. Jaderberg, D. Hassabis, J. M. Jumper, *Nature* **2024**, *630*, 493-500.
[13] E. C. Meng, T. D. Goddard, E. F. Pettersen, G. S. Couch, Z. J. Pearson, J. H. Morris, T. E. Ferrin, *Protein Sci.* **2023**, *32*, e4792.
[14] S. Passaro, G. Corso, J. Wohlwend, M. Reveiz, S. Thaler, V. R. Somnath, N. Getz, T. Portnoi, J. Roy, H. Stark, D. Kwabi-Addo, D. Beaini, T. Jaakkola, R. Barzilay, *bioRxiv* **2025**, 2025.2006.2014.659707.
[15] C. A. K. Lundgren, D. Sjostrand, O. Biner, M. Bennett, A. Rudling, A. L. Johansson, P. Brzezinski, J. Carlsson, C. von Ballmoos, M. Hogbom, *Nat. Chem. Biol.* **2018**, *14*, 788-793.
[16] S. K. Small, M. R. O'Brian, *J. Bacteriol.* **2011**, *193*, 4088-4094.
[17] P. Skotnicová, R. Sobotka, M. Shepherd, J. Hájek, P. Hrouzek, M. Tichý, *J. Biol. Chem.* **2018**, *293*, 12394-12404.
[18] B. Svensson, L. Hederstedt, *J. Bacteriol.* **1994**, *176*, 6663-6671.
[19] R. Cross, J. Aish, S. J. Paston, R. K. Poole, J. W. Moir, *J. Bacteriol.* **2000**, *182*, 1442-1447.
[20] X. Robert, P. Gouet, *Nucleic Acids Res.* **2014**, *42*, W320-W324.
[21] T. Stiernagle, *WormBook* **2006**, http://www.wormbook.org.
[22] I. Richter, S. Radosa, Z. Cseresnyés, I. Ferling, H. Büttner, S. P. Niehs, R. Gerst, K. Scherlach, M. T. Figge, F. Hillmann, C. Hertweck, *mBio* **2022**, *13*, e01440-01422.
[23] F. Trottmann, K. Ishida, M. Ishida-Ito, H. Kries, M. Groll, C. Hertweck, *Nat. Chem.* **2022**, *14*, 884-890.
[24] F. Trottmann, J. Fiedler, K. Ishida, M. Ishida-Ito, R. F. Little, C. Hertweck, *ACS Chem. Biol.* **2023**, *18*, 1557-1563.

Author contributions

J. F. preparation of *Pbur*Δ*burK* and *Pbur*Δ*burK* pSCrhaB2-*burK*, primer design, genetic complementation of *B. thailandensis* *Pbur*Δ*burK* with *burK* homologs, HPLC-HRMS^2 analyses, conceptualization, visualization, research design, data compilation, writing.

I. R. nematode assays, microscopic analyses, figure design, writing.

K. D. site-directed mutagenesis of *burK*, genetic complementation of *B. thailandensis Pbur*Δ*burK* with point mutation variants, proof-reading.

A. S. cloning of pET28-*burK*, whole-cell assays using recombinant production of BurK, proof-reading.

C. H. Research design, conceptualization, supervision, writing, figure design, funding acquisition, resources.
